# Advanced immunotherapies for glioblastoma: tumor neoantigen vaccines in combination with immunomodulators

**DOI:** 10.1186/s40478-023-01569-y

**Published:** 2023-05-10

**Authors:** Berta Segura-Collar, Sara Hiller-Vallina, Olaya de Dios, Marta Caamaño-Moreno, Lucia Mondejar-Ruescas, Juan M. Sepulveda-Sanchez, Ricardo Gargini

**Affiliations:** 1 Instituto de Investigaciones Biomédicas I+12, Hospital Universitario, 12 de Octubre, 28041 Madrid, Spain; 2 Pathology and Neurooncology Unit, Hospital Universitario, 12 de Octubre, Av. de Córdoba, S/N, 28041 Madrid, Spain; 3 Instituto de Salud Carlos III, UFIEC, 28222 Majadahonda, Spain; 4 Medical Oncology, Hospital Universitario, 12 de Octubre, 28041 Madrid, Spain

**Keywords:** Glioblastoma, Glioma, Neoantigen vaccine, mRNA vaccine, Virotherapy, Immunotherapy, Tumor microenvironment, Suppressive myeloid cells, Immune checkpoint inhibitors, PD-1, PD-L1

## Abstract

Glial-origin brain tumors, including glioblastomas (GBM), have one of the worst prognoses due to their rapid and fatal progression. From an oncological point of view, advances in complete surgical resection fail to eliminate the entire tumor and the remaining cells allow a rapid recurrence, which does not respond to traditional therapeutic treatments. Here, we have reviewed new immunotherapy strategies in association with the knowledge of the immune micro-environment. To understand the best lines for the future, we address the advances in the design of neoantigen vaccines and possible new immune modulators. Recently, the efficacy and availability of vaccine development with different formulations, especially liposome plus mRNA vaccines, has been observed. We believe that the application of new strategies used with mRNA vaccines in combination with personalized medicine (guided by different omic’s strategies) could give good results in glioma therapy. In addition, a large part of the possible advances in new immunotherapy strategies focused on GBM may be key improving current therapies of immune checkpoint inhibitors (ICI), given the fact that this type of tumor has been highly refractory to ICI.

## Background (introduction)

Gliomas are the most common primary brain tumor, glioblastoma (grade IV glioma, IDH wt) is the most aggressive type of cancer with one of the worst prognoses, due to the lack of effective therapies [37]. Diffuse gliomas are histologically classified as low and intermediate-grade gliomas (grades II and III) (herein called Lower-Grade Gliomas, LGG) or glioblastomas (GBMs) (grade IV gliomas). GBM one of the most aggressive cancers of the central nervous system, presenting only 5% of patient with 5-year survival rate. The histological features of these aggressive gliomas include high cellularity, nuclear atypia, microvascular proliferation, brisk mitotic activity, and frequent areas of necrosis [75]. New therapeutic strategies for gliomas, including kinase inhibitors, alkylating agents, proteasome inhibitors and transcription factor inhibitors have failed to improve overall survival in the last 20 years. Even the therapeutic advances with immunotherapy have not given good results. Recently clinical trials with inhibitors for PD-1 in recurrent and newly diagnosed glioblastoma have not shown an improvement in patient survival [102]. Consequently, a large collection of articles has characterized the glioma microenvironment to understand the cellular complexity that makes these tumors the most difficult to treat. Initially, the focus was on vascular, but more recently there has been an increasing interest in the immune cell components of the microenvironment [3, 104]. Glioma belongs to the group of tumors called cold tumors; a group characterized by the low content of immune cells [120]. However, this is not entirely correct since despite having a low proportion of lymphocytes, they show a strong infiltration of myeloid cells, both brain resident myeloid cells (microglia) and peripherally recruited macrophages and MDSCs. Thus, this type of tumor exhibits a strongly immunosuppressed myeloid landscape that also enhances the proliferation of tumor cells [97, 99]. This is due to a large proportion of the immune cells that make up the glioma microenvironment are macrophages/microglia with immune-suppressing properties, such as M2 macrophages and myeloid-derived suppressor cells (MDSCs) [42, 43].

## Brain: an organ with a special immunological environment

The anatomical location of the brain provides robust protection toward the outside of the against external factor. Unless there is an injury or they escape the specific innate and adaptive immune defense mechanisms, foreign material or pathogens are quite unlikely to directly reach the brain. Moreover, the brain resides behind blood–brain barriers (BBBs) and blood-cerebrospinal fluid barrier (BCSFB) that restrict pathogen and immune cell entry from the periphery into the parenchyma. Thus, the brain has a unique relationship with the immune system unlike the rest of the peripheral organs.

Conceptually, the term ‘immune privilege organs’ refers to these organs, in which experimentally implanted tissue grafts are incapable of provoking immunity leading to graft rejection. In the case of the brain, these experiments are habitually attributed to Peter Medawar [85], who showed a readily rejection to foreign tissues when grafted into peripheral sites like the skin, but when grafted into the brain parenchyma, they survived for prolonged durations. Based on these observations and others, emerged the idea that antigens contained within the brain could not be seen by the immune system as lack of conventional lymphatic vessels in the brain and that parenchyma would prohibit the drainage of brain antigens into peripheral lymphatic tissues, parallel the BBB inhibit immune cell entry into the brain [30].

Subsequent observations showed that cerebrospinal fluid (CSF), which contains solutes and immune cells, drains into cervical lymph nodes. Moreover, activated circulating T cells can cross the BBB and overcome immunosurveillance within the brain, even in the absence of neuroinflammation [76]. Therefore, the view about the BBB action as a ‘hermetic seal’ to immune cell entry, has changed. Equally, damage incurred to the BBB in the context of gliomas and other tumors limits the restrictions normally proffered by the BBB [134]. The relevance of BBB dysfunction in the development of CNS pathologies is widely documented and associated with neuroinflammation processes [108]. In particular, the alteration of the BBB has special relevance in the pathology of the glioma and in the infiltration of immune cells in the microenvironment of the glioma in both, high and low grade [15, 107].

In this concept, it has recently been proposed that the brain is not an immune-privileged organ but simply has a specific morphological architecture that generates different immune responses compared to peripheral sites [30]. The brain parenchyma allows prioritizing the proper function of neurons over eliciting an immune response. While ventricular spaces and border compartments (subarachnoid and perivascular spaces) are dedicated to brain immunity [14]. Within parenchyma, macrophages serve as guards collecting all the information from the brain, which they can present to the immune-surveying T cells. If T cells recognize their specific antigen during communication with macrophages, they will become activated and will be allowed the entry of additional immune cells into the brain [100], nevertheless in the absence of it, T cells were confined to this space.

In fact, as two articles have described, the special structuring of the CNS particularly affects the coordination of the immune system response to CNS infections [84, 96]. All of this suggests that the CNS has a specialized immunological environment, but it may not be a privileged one.

## Immune response modulators in the tumor microenvironment (TME)

The process of tumorigenesis requires a crosstalk with the tissue where it develops, in which a direct interaction is generated with specific cells of healthy tissue and tumor cells [37]. For some time now, the importance of the microenvironment in the development of tumor processes has been recognized, as it plays a crucial part in obtaining nutrients through the various processes of angiogenesis and also in the various tumor mechanisms of immune evasion [46].

GBM is associated with marked local and systemic immunosuppression. In fact, a distinctive feature of GBM is the development of a deeply immunosuppressive TME and cold phenotype, which can stop endogenous antitumor immune responses and limiting the effectiveness of immunotherapy [31, 121], making the TME unique in its composition.

**Fig. 1:**
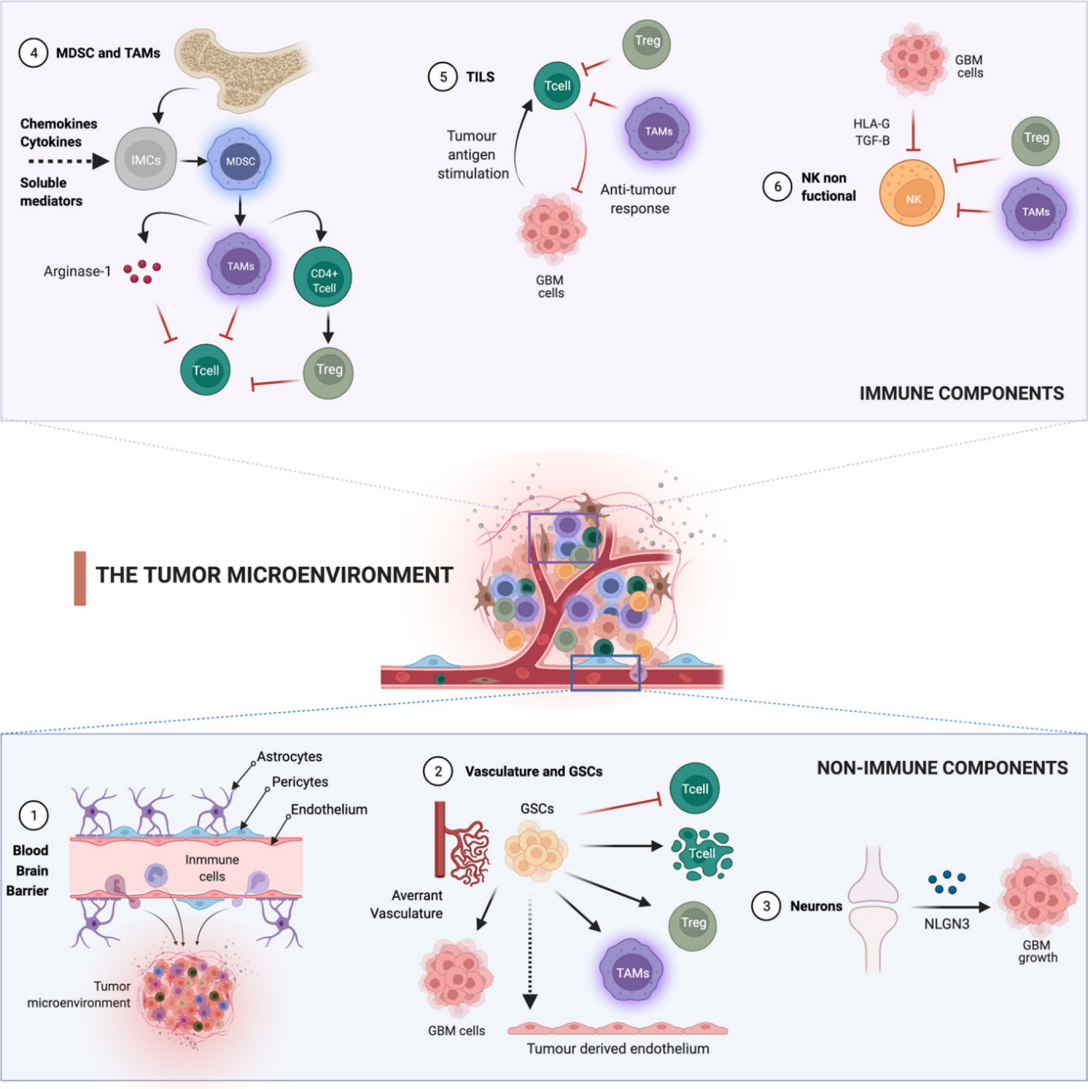
Immune and non-immune components of the glioma microenvironment. (1) Blood–Brain Barrier (BBB). The BBB’s main components are endothelial cells, pericytes, and astrocytes. In the GBM the BBB is disrupted and there is an active efflux of immune cells into the TME and the astrocytes support tumor growth by the secretion of growth factors and cytokines. (2) Vasculature and Glioma Stem Cells (GSCs). An aberrant vasculature and hypoxia support the TME. The GSCs can differentiate into endothelial cells generating the glioma vasculature. GSCs also can directly inhibit T cell proliferation and activation, trigger T cell apoptosis, induce T regulatory cells (Tregs) and immunosuppressive characteristics of tumor-associated microglia/macrophages (TAMs) induction. (3) Neurons. Post-synaptic neurons support the progression of gliomas by mitogenic to neoplastic cells through the upregulation of neuroligin-3 (NLGN3). (4) Myeloid-Derived Suppressor Cells (MDSC) and TAMs. Multiple chemokines and soluble mediators secreted by gliomas initiate immunosuppressive pathways that commit immature myeloid cells (IMCs) to become MDSCs and promote the differentiation of MDSCs towards TAMs. TAMs inhibit T-cell by the production of Arginase-1, anti-inflammatory cytokines production, and when TAMs release low levels of IFN-γ and high levels of IL-10, microglia act as potent Tregs inducers. (5) Tumor-infiltrating lymphocytes (TILs). TILs have an ineffective immune response because of the inhibitory action of Tregs, MDSC, TAMs, and the expression of some molecules by GBM cells that suppress the lytic action of lymphocytes. (6) Natural Killers (NK). NKs are non-functional because of the direct action of TAMs, MDSCs, and Tregs, and the expression of HLA-G by the GBM cells and TGF-β, which act as inhibitory ligands for activated NK cells. Figure Created with BioRender.com

The GBM microenvironment shares some characteristics with tumors that have responded to immunotherapy but are unique for the resident cells of the CNS. In addition, the BBB contributes to the brain being considered an immunologically privileged organ with very precise regulation of immune responses, leading to a more immunosuppressive environment [121]. Next, we will try to detail the non-immune and immune components of the TME in the GBM (Fig. 1).

### Non-immune cell components

Several stromal non-immune components promote the immunosuppression originating around the tumor cells.

*Blood–Brain Barrier (BBB)* The BBB is a unique component of the brain that allows for the maintenance of homeostasis in the CNS by forming a tightly regulated neurovascular unit that includes endothelial cells, pericytes, and astrocytes [3]. In the GBM TME, the BBB is disrupted and displays increased and heterogeneous permeability with an active efflux of immune cells into the TME, resulting in a vasculature known as the blood–tumor barrier (BTB) [3].

*Astrocytes* The astrocytes are essential for a proper healthy brain function. Among their functions, they provide structural support in the brain through the maintenance of homeostasis, having a key role in the preservation of BBB [136]. In addition, in a healthy CNS, astrocytes react to CNS injury through the secretion of growth factors and cytokines to enable the repair of brain tissue [112]. However, in the TME this process, known as astrogliosis or reactive gliosis, can support tumor growth and mediates resistance to glioma therapy [98]_._ This disjunction defines the existence of different astrocytic phenotypes depending on the cells and the surrounding microenvironment. On the one hand, the "A2" astrocyte is associated with an anti-inflammatory environment that would promote repair in response to damage by seeking homeostasis in the brain. On the other hand, the "A1" astrocyte responds to pro-inflammatory stimuli and is related to mounting an immune response such as antigen presentation or complement activation markers. Specifically, the GBM tumor environment promotes the interaction of the astrocyte with the surrounding microglia, leading to the upregulation of the JAK/STAT and PD-L1 pathway in astrocytes. Activation of these pathways leads to increased levels of anti-inflammatory cytokines such as IL-10, TGF-β, and STAT3 contributing to the immunosuppressive environment and maintenance of the cold tumour environment [49].

*Vasculature* An aberrant vasculature and atypical organization are characteristics of the GBM microenvironment where edema, hypoxia, and necrosis are frequently observed. Hypoxia is a common feature of solid tumors that can activate angiogenesis, increase the survival of tumors, as well as suppress anti-tumor immunity and hinder the therapeutic response [29].

*Glioma Stem Cells (GSCs)* GSCs refer to a population of tumor-originating cells capable of self-renewal and differentiation [93]. These cells are located within perivascular areas of the TME, which suggests that the GSCs are able to differentiate into endothelial cells, generating the glioma vasculature and giving rise to GSCs deposits in the perivascular niche [17]. GSCs also have a role in immunomodulatory reaction to GBM and can directly inhibit T-cell proliferation and activation, besides inducing T regulatory cells (Tregs), and trigger T-cell apoptosis [8]. In addition, GSCs can influence innate immunity by inducing immunosuppressive characteristics of tumor-associated microglia/macrophages (TAMs) [136]. Therefore, GSCs contribute to the growth of GBM cells and the development of resistance to treatments through the induction of an immunosuppressive TME.

*Neurons* The neurons are excitable and specific cells of nervous tissue. In the TME, post-synaptic neurons support the progression of gliomas by promoting the transition of mitogenic to neoplastic cells through the upregulation of neuroligin-3 (NLGN3), inducing a phosphoinositide 3-kinase (PI3K) signaling-mediated proliferative activity in glioma cells [73]. In humans, it has been demonstrated that NLGN3 expression in GBM is inversely correlated with patient survival [129]. In this sense several studies have shown that gliomas are robustly regulated by neuronal activity. The findings of Monje M. and Winkler F. Lab indicate that synaptic and electrical integration in neural circuits promotes glioma progression. They demonstrated that neuron-glioma interaction include communication synapse-dependent mediated by glutamate receptors of the AMPA subtype [128], and non-synaptic dependent through membrane depolarization by potassium currents [128], both of which promote glioma proliferation.

### Immune cells

*MDSCs and TAMs* MDSCs and TAMs are a heterogeneous population of immature myeloid cells, that are located in the tumor tissue and the peripheral blood of patients with glioma. Although TAMs and MDSCs are considered separate entities, the boundaries between them are not clearly delimited and they share the expression of common markers and perform similar functions [127]. MDSCs share some common features such as their myeloid origin, immature state, and most importantly, the ability to convert immune responses from a Th1 phenotype toward a Th2 phenotype. This conversion results in potent inhibition of CD4^+^ and CD8^+^ T-cells and significant immunosuppression in tumor settings [80]. An extensive MDSC infiltration around the TME has been observed in all glioma models and patients. Multiple chemokines (pro-inflammatory factors, activated T-cell-derived cytokines) and soluble mediators secreted by gliomas, attract MDSCs towards the tumor and synergistically initiate immunosuppressive pathways that commit immature myeloid cells to become MDSCs, a process that further promotes the differentiation of MDSCs toward TAMs [80]_._

BMDM are a set of TAM populations who are recruited from circulating bone marrow-derived macrophages/monocytes (BMDM) [9]. Both subtypes are mononuclear cells that can inhibit CD4 and CD8 lymphocytes. It is known that, in the TME of GBM, TAMs have pro-tumoral roles (M2 TAMs), and their accumulation is related to the tumoral grade [140]. The current evidence shows that TAMs support the growth and invasion of glioma cells through different mechanisms [22, 80]

- The degradation of l-arginine, a molecule essential for the proliferation and activation of T-cell, by the production of Arginase-1.
- The reduction of the migration and infiltration of immune cells by anti-inflammatory cytokines production.
- The release low levels of IFN-γ and high levels of IL-10, microglia acts as potent TAMs inducers and supporting the immune suppression in the glioma environment.

Thus, TAMs have a wide range of immunosuppressive functions being an important contributor to the immunosuppressive TME in gliomas.

Microglia represent brain intrinsic macrophages which are activated locally by the TME. The identification and tracking of BMDMs and microglia have been problematic due to the lack of consistent markers. Recent studies suggest that the main difference between these two populations is their location in the tumor. They have shown that in the tumor core there is predominantly BMDMs while in tumor periphery they are mostly microglia [67]. Microglia are also described in terms of their activation status, often classified as M1 with antitumor effects or M2 with pro-tumor effects, the same as BMDMs. In this sense, the latest studies also propose that the functionality of these two subpopulations of TAMs does not depend per se on which subpopulation they belong to, but on its location linked to the signals it perceives from the TME. Tumor core macrophages evolve towards a pro-inflammatory state while those at the periphery evolve towards an anti-inflammatory state [67].

*Tumor-infiltrating lymphocytes (TILs)* TILs are present in the TME of GBM, including CD4^+^ and CD8^+^ T-cells, and CD4^+^CD25^+^FoxP3^+^ Tregs [8]. However, TILs often have a dysfunctional, exhausted phenotype that renders them ineffective in their immune responses. This suppressive action is carried out by TGF-β [23], IL-10 cytokine [52], and CCL2 (MCP-1) [117], which are released by the glioma and microenvironmental cells, which recruits Tregs, MDSC, and TAMs infiltrating the tumor disrupting lymphocyte function. Glioma cells also express molecules that suppress the lytic action of lymphocytes, such us FAS ligand (FasL) [109] PD-L1 and PD-L2 [63].Specifically, the immunological synapse generated by the PD-1/PD-L1 interaction is a key point and important object of study today.

The absence of TILs is one of the main factors involved in the lack of response or resistance to immunotherapy. Moreover, recent data suggest that while high TILs infiltration is associated with better outcome overall, only immune infiltrates expressing PD-1 and PD-L1 appear to be relevant in the response to immune checkpoint inhibitors [113]. Additionally, exhausted T cells accumulate in TME results in resistance and relapse in CAR-T cell therapy [81].

*Natural Killers (NK)* NKs are innate lymphoid cells that represent around 10% of all circulating lymphocytes [65] and are activated against tumor cells in different neoplasms. In GBM, NKs are a minor component of TME, comprising only about 2% of the cells of the immune infiltrate. Unfortunately, the infiltrating NK cells in GBM have been found to be non-functional, which can be attributed to the following causes:

The direct contact with other immunosuppressive cells such as TAMs, MDSCs, and Tregs [8].

- The expression of the inhibitory ligands HLA-G by the GBM cells and the anti-inflammatory cytokine TGF-β, which act as inhibitory signal for activated NK cell [111, 135].

A human study shows a decrease in NK levels in GBM patients compared to healthy control [71].

Given the complexity and relevance of the tumor microenvironment in the immunosuppressive phenotype, it is crucial to consider it in the development of new therapies. Concept that will help to understand why GBM behaves differently from other cancers and develop new immunotherapy strategies to target tumor cells more precisely.

## Implication of tumor mutational burden (TMB) in therapeutic efficacy

Cancer immunotherapy relies on the immune system's ability to target specific tumor antigens and generate a response [106]. T cells normally recognize neoantigens, produced by mutations, which are presented by major histocompatibility complex (MHC) proteins on the surface of cancer cells, and target these cells for destruction [56]. To avoid the host’s immune response, tumor cells express cell-surface proteins able to interact with “checkpoint” proteins expressed on immune cells [91]. The checkpoint proteins are an important regulatory component necessary for suppressing immune responses after threat elimination, however in this case the cancer cells use this mechanism to inactivate immune cells before they can detect and eliminate them [91].

Immunotherapy with immune checkpoint inhibitors (ICIs) works by blocking immune checkpoint proteins avoiding the inactivation of T cells [91]. Currently, an inhibitor of the T-lymphocyte-associate antigen 4 (CTLA-4) and six inhibitors of the programmed cell death protein pathway (PD-1/PD-L1) have received regulatory approval from US Food and Drug Administration (FDA) for different cancer types, but not in gliomas [126].

To date, numerous markers with possible prognostic value have been described, such as/including cytokine levels, tumor cell antigens, delayed-type hypersensitivity reactions, PD-L1 expression, or tumor mutational load (TMB), with the last two biomarkers being particularly relevant.

*PD-L1 expression* The expression of PD-L1, measured by immunohistochemistry (IHC) is one of the most important response markers to ICIS but at the same time has multiple limitations, including the technical issues and the variability of response [56]. It is noteworthy that the antibody used to measure PD-L1 expression can greatly impact on the positive rate and subcellular distribution of PD-L1 in glioma cells [18]. Regarding its prognostic value, although some reports have showed a lack of association, numerous studies have concluded that expression of PD-L1 is a good biomarker associated with worse evolution and overall survival (Table 1).

**Table 1:** PD-L1 expression and tumor mutational burden in the prognosis of glioma patient

Authors (Ref)	Year	Marker	Prognostic value
Berghoff A et al. [5]	2015	PD-L1	No significant differences in PD-L1 between initial and recurrent GBM specimens or with patient outcomes
Zeng et al. [142]	2016	PD-L1	No significant relation between PD-L1 expression and OS, but a strong tendency. PD-L1 expression was significantly associated with poor OS in the patients with long-time survival or follow-up (OS ≥ 12 months)
Jan et al. [55]	2018	PD-L1	No significant relation between PD-L1 expression and the prognostic factors OS and PFS
Knudsen et al. [66]	2021	PD-L1	PD-L1 was expressed in all investigated GBMs but didn’t show prognostic value
Nduom E et al. [88]	2016	PD-L1	Higher expression of PD-L1 correlated significantly with worse outcomes
Wang et al. [133]	2016	PD-L1	Higher expression of PD-L1 indicated significantly shorter survival, especially in GBM
Han et al. [45]	2017	PD-L1	High expression of PD-L1 in tumor cells was an independent and significant predictive factor for worse OS
Xue et al. [138]	2017	PD-L1	High PD-L1 expression was associated with worse OS in glioma and GBM patients
Bloch O Et al. [6]	2017	PD-L1	PD-L1 expression was the primary independent predictor of survival
Lee et al. [70]	2018	PD-L1	PD-L1 expression in tumor cells was significantly associated with poor OS, though multivariate Cox analysis did not confirm this association. PD-L1 target therapy might be beneficial for PD-L1-expressing GBM patients with poor prognosis
Pratt D et al. [95]	2018	PD-L1	A 5% PD-L1 expression cut-off identified a subset of glioblastoma associated with a worse clinical outcome
Samstein R et al. [105]	2019	TMB	No association between higher TMB and improved survival in patients with glioma
Zhao J et al. [144]	2019	TMB	No significant inverse relationship between TMB and radiographic/histological responses to PD1 blockade was observed in a recurrent GBM patient cohort
Touat M et al. [123]	2020	TMB	Hypermutant gliomas with mismatch repair (MMR) deficiency are less responsive to PD1 blockade than gliomas with lower TMB
Yin W et al. [141]	2020	TMB	TMB was not an independent prognostic factor in LGG, but the TMB-related immune-related risk score was
Draaisma K et al. [27]	2015	TMB	Tumor grade was correlated with the TMB: grade II diffuse gliomas had fewer genetic changes than grade III or IV
Wang L et al. [132]	2020	TMB	Patients with a higher TMB exhibited shorter overall survival, being an independent prognostic factor for glioma
Gromeier M et al. [40]	2021	TMB	Very low TMB is associated with longer survival after ICIs in recurrent glioblastoma patients, while it is not observed in cohorts of immunotherapy naïve newly diagnosed or recurrent glioblastoma patients without ICIs
Hodges T et al. [51]	2017	TMB and PD-L1	Biomarkers are expressed infrequently in GBM without substantial overlap

*OS*, overall survival; *PFS*, progression-free survival; *MMR*, mismatch repair; *ICIs*, immune checkpoint inhibitors

*Tumor mutational burden (TMB)* TMB reflects the cancer mutation quantity [56], which means the number of non-inherited mutations per million bases of investigated genomic sequence [87]. High TMB may be a consequence of a deficiency/mutation in DNA repair genes, such as the mismatch repair (MMR) or DNA Polymerase ε (POLE) mutation. Another possible cause of TMB might be the exposure to agents able to promote DNA damage, i.e., cancer risks factors (smoke or radiation) or anti-cancer agents (alkylating agents) [26].

Since TMB was associated with the presence of a greater number of tumor-neoantigens, which facilitated immunological recognition and the development of antitumor immune response, TMB has been proposed as a possible predictive marker of response to ICIs in solid tumors [56]. Samstein R et al*. *[105] demonstrated from a large cohort of patients treated with ICIs that TMB can predict survival across diverse types of human cancers, being relevant in patients treated with either anti-CTLA-4 or anti-PD-1 therapies [105]. However, unlike cancer types, there was no association between higher TMB and improved survival in patients with glioma; in fact, the trend was towards a poorer survival [105]. In this sense it is important to note that TMB is generally low in gliomas compared to other tumors [1]. Moreover, currently there is not enough evidence demonstrating the prognostic value of the TMB variable and its role remains unclear (Table 1).

## Immunotherapeutic approaches for gliomas

### Immunocheckpoint inhibitors

Despite the promising results with the blockade of the PD-1/PD-L1 axis or CTLA4 in some solid cancers, no successful results have been obtained in GBM. There are several clinical trials studying the anti-PD1 therapy, such as nivolumab, either to evaluate patient survival compared to other treatments such as bevacizumab (anti-VEGFA) (CheckMate-143) or the standard combination of radiation and TMZ (NCT02617589). Disappointing results from these trials have shifted the focus of this therapy to the search for etiologic factors contributing to treatment failures.

**Fig. 2:**
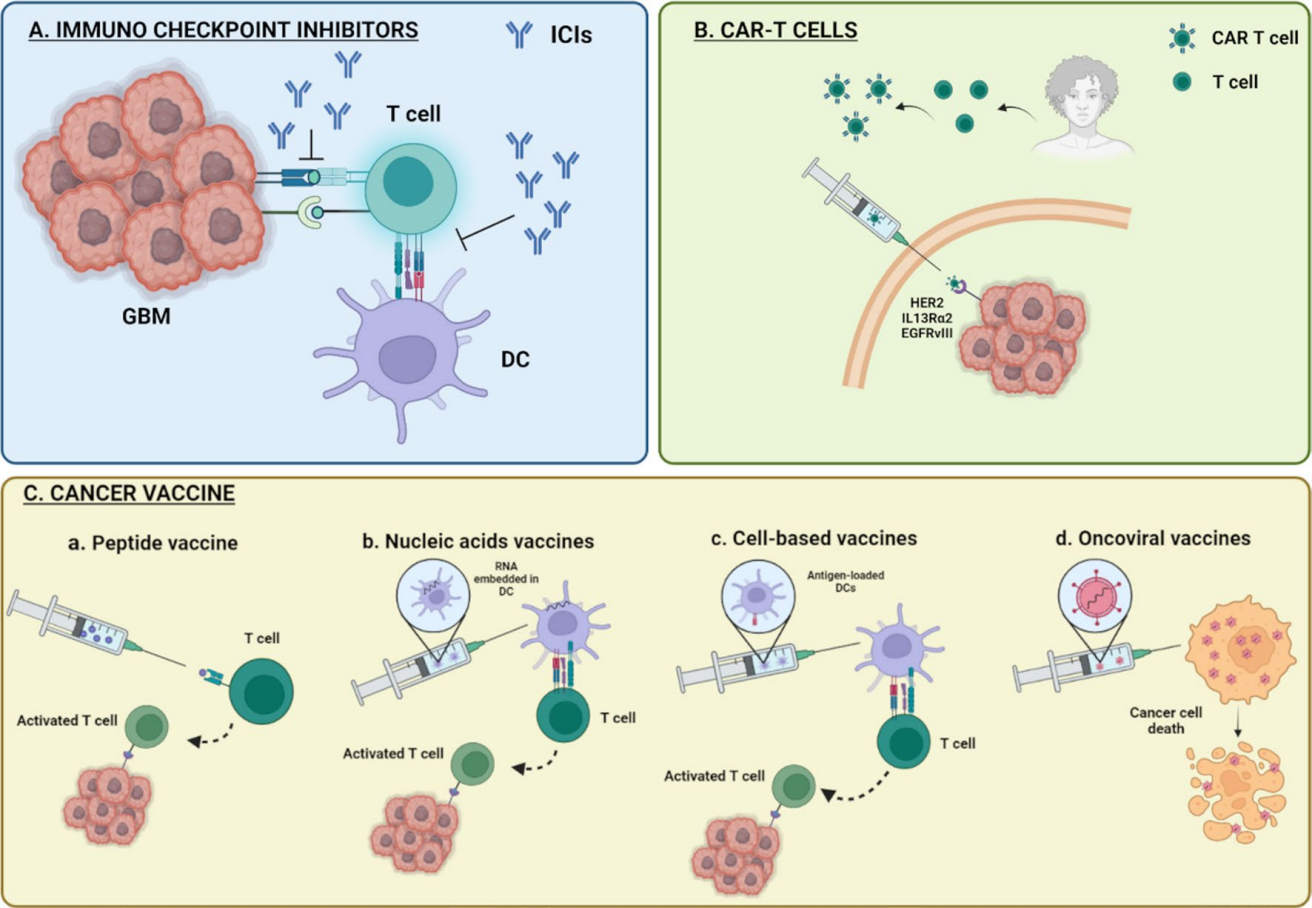
Current mechanisms underlying immunotherapeutic approaches for gliomas. **A** Immunocheckpoints inhibitors (ICIs). Immunotherapy approach based on glioblastoma (GBM) expressed immunomodulatory co-receptor (immunocheckpoint)—T cell interaction blockade, thereby causing the inhibition of the immunosuppression. **B** CAR T cell. Autologous transplant with in vitro modified specific T cell against tumor neoantigens. **C** Cancer vaccines. Vaccines stimulating an adaptive immune system response. *a*. Peptide vaccine. Anti-tumor response activation by exogenously administrated peptides, which recognizes tumor-specific neoantigens. *b*. Activation of tumor immune response by synthesized nucleic acid sequence for a specific antigen. *c*. Cell-based vaccines. Autologous transplant of antigen-presenting cells loaded with tumor-specific antigen resulting in MHC-mediated T cell presentation, activating anti-tumor response. *d*. Oncoviral vaccines. Therapy based on virus high replication rate and their lytic capacity

Inherent obstacles to immune checkpoint blockade in glioblastoma may be due to the wide intratumorally heterogeneity or restricted access of drugs and immune cells to the CNS. On this matter, it is unclear whether PD1- and CTLA4-blocking antibodies must be positioned within tumors for activity, rather than simply acting on peripheral T cells prior to entry into the CNS. Likewise, another difficulty for the efficacy of this therapeutic treatment in GBM is the T cells exhaustion and the presence of alternative immune checkpoints, such as TIM3, LAG3, BTLA, CD244, CD160, CD39 or TIGIT, which lead to a state of terminal exhaustion that cannot be reversed solely by traditional immune checkpoint blockade (Fig. 2). On this point, clinical trials in GBM patients are currently underway, targeting TIM3 and LAG3 alone or in combination with anti-PD-1 therapy (NCT02658981 and NCT02817633).

### CARs and adoptive cell transfer

This type of therapy is based on the use of modified immune cells as a vehicle. T cells Chimeric Antigen Receptors (CAR T cells) are proteins that have an extracellular ligand-recognition domain, transmembrane domain, and intracellular signaling domain that induces T cell activation [139]. It is based on passive immunotherapy where T cells are extracted from the individual, charged with the CAR molecule, and presented with tumor antigens. Once clonal expansion occurs, they are introduced into the individual to create an anti-tumor response (Fig. 2).

For the treatment of GBM, clinical trial results are available for CAR T cells targeting three anti-gens: EGFRvIII, human epidermal growth factor receptor 2 (HER2; also known as ERBB2) and IL-13 receptor α2 (IL-13Rα2) [104]. These trials have demonstrated that the use of CAR T cells for brain tumors is feasible, safe and potentially efficacious. As occurs in other solid tumors, the use of CAR T cells for brain tumors still faces several substantial obstacles. One major problem is the heterogeneous expression of target antigens in tumor cells. Even in the case of uniformly expressed antigens, selective pressure can result in antigen loss and tumor recurrence. In the first clinical trial of EGFRvIII-directed CAR T cells for GBM, a significant decrease in EGFRvIII expression was demonstrated in almost all patients in which tumor-infiltrating CAR T cells were detected, but not in wild-type EGFR [89]. In one patient with recurrent multifocal glioblastoma, intracranial administration of IL-13Rα2-targeted CAR T cells resulted in the regression of all intracranial and spinal lesions, but subsequent relapse was in IL-13Rα2-negative tumors [10]. This suggests that successful CAR T cell therapy will require either targeting multiple antigens or the development of CAR T cell designs that induce significant epitope spreading. Any of the mentioned approach would lead to a broader immune response, which might also carry the risk of unintended reactivity against normal tissue.

Recently, NK cells have received much attention as alternative CAR-engineered effectors for the treatment of glioblastoma [12]. NK cells are not only involved in antitumor immunity by eliminating malignant cells but also regulate tumor-specific adaptive immune responses through crosstalk with dendritic cells (DCs). The principal advantage of CAR-NK cells for cancer treatment is the capacity to eradicate cancer cells not only in a CAR-dependent manner, but also in a CAR-independent manner [137]. CAR-NK cells naturally exhibit cytotoxic activity through CAR-independent receptors that are expressed by tumor cells, which may help to eradicate glioblastoma cells with low or heterogeneous expression of the CAR target tumor-associated antigens (TAA). Clinical studies on the treatment of malignant glioma using CAR T cells and CAR NK cells are in progress. However, there was a study on CAR NK cells transduced with bispecific CAR constructs as a solution to antigen loss in EGFRvIII-directed CAR NK cell therapy for glioblastoma, targeting both mutated and wild-type EGFR. Intratumorally injections prolonged the survival of glioblastoma xenograft mouse models without antigen escape [44].

The problem with this therapeutic approach is the immune suppression of glioma and the low immunogenicity of the tumor. Therefore, it is necessary to find new highly immunogenic neoantigens and surpass TEM suppression. Adoptive T cell therapy holds considerable promise for the treatment of brain tumors. While The administration of autologous TILs has induced regressions in some tumor types, it is less feasible in glioblastoma, owing the difficulty of isolating and expanding TILs from the CNS.

### Cancer vaccine

Cancer vaccines are a type of active immunotherapy aimed at stimulating the patient's adaptive immune system against specific TAAs to induce tumor regression and to have long-lasting memory responses to prevent tumor recurrence. Currently, various types of vaccines are being used for the treatment of gliomas (Fig. 2).

#### Peptide vaccines

Peptide vaccines consist of exogenous administration of Tumor-specific Antigens (TSA) to induce the response of the adaptive immune system (Fig. 2). So far, vaccines directed against a single tumor antigen have shown limited efficacy due to the heterogeneity of glioblastoma. This is the case of anti EGFRvIII ridopepimut vaccine (CDX-110), which is not showing significant improvements in the vaccinated group of patients with respect to the control group in phase III clinical trial. These data suggest that future peptide vaccines could be directed against multiple antigens.

Within this group of vaccines, the vaccine against the R132H mutation of IDH1 tested in grade III and IV gliomas presenting this mutation, is noteworthy. The results obtained in phase I clinical trial (NCT02454634) have shown a 3-year progression-free and death-free rate of 0.63 and 0.84, respectively.

#### Nucleic acids vaccines

These vaccines are based on using these molecules to express the protein and produce an immune response. Several studies advocate the use of one or the other, but in recent times, it has been seen that the RNA vaccine appears to be the most effective and safe one, it can include mRNA, siRNA and miRNA. RNA can be introduced into the organism in various ways, but since it cannot be integrated into the host’s DNA, as it does not have the ability to self-replicate, and it is rapidly degraded by RNases. This is a great handicap for this type of vaccine since it is a very unstable material and has a short period of lifespan. In addition, by translating into immune cells, there is no longer HLA restriction in patients, producing a specific antigen-T cell response. These vaccines can be introduced by modified autologous DCs, or by nanoparticles such as liposomes or dendrimers that are captured in the ganglia (Fig. 2). In addition, RNA is an agonist of the Toll-like receptors (TLR) which produces the activation of these mediating innate immune responses against the tumor, synergizing with the adaptive responses produced by the APCs [86].

#### Cell-based vaccines

DC vaccination aims to address the often failure of peptide vaccine, even when conjunction with immunostimulatory adjuvants, trying to reverse the immune system ignorance to TAA or TSA cells. To achieve this, the DCs are stimulated for maturation and loaded with tumor-associated peptide antigens on their MHC molecules ex vivo. The Generation of DC vaccine for cancer therapy involves several steps: First, isolating CD14^+^ monocytes from patient PBMCs. These monocytes are cultured on granulocyte–macrophage colony-stimulating factor (GM-CSF) and IL-4 for 5–7 days to differentiate into immature DCs. Then, for differentiation of immature DCs into mature DCs, immature DCs are incubated for 16–20 h in a cytokine cocktail with GM-CSF, IL-4, TNFα, IL-1β, and IL-6, in this point, the DCs are loaded with TAAs or TSAs. DCs uptake and process these antigens and present epitopes on their MHC molecules at the cell surface. Finally, these mature antigen-loaded DCs are then injected back into the patient (Fig. 2) [28].

Therefore, there have been some clinical trials of DC immunotherapy for GBM. In 2012, Ardon et al*.* [2] reported a phase I/II clinical trial that enrolled 77 patients with newly diagnosed glioblastoma. In that study, four weekly induction autologous glioblastoma lysate-loaded DC vaccines were administered intradermally to glioblastoma patients after radiotherapy, but before maintenance chemotherapy with temozolomide. The results showed a median overall survival of 18.3 months in the treated group. A recent phase III clinical trial of an autologous tumor lysate-pulsed DCs vaccines in patients with newly diagnosed glioblastomas showed extended patient survival [72]. The authors reported that the median overall survival of treated group was 23.1 months from the time of surgery, with 2- and 3-year survival rates of 46.2% and 25.4%, respectively.

#### Oncoviral vaccines

The use of modified viruses has spread in recent years, paving the way for their use as oncotherapy. Viruses are not only able to induce a response to a specific tumor antigen, but they also activate the immune system by themselves, producing an innate response. In addition, there is a susceptibility of gliomas to viral infections due to the loss of antiviral phenotype in malignant cells. All this considerably increases the effect and effectiveness of vaccines. Generally, oncolytic viruses only can selectively replicate in tumor cells, producing cell lysis and antigen presentation (Fig. 2). The most commonly viruses used in clinical trials involving patients presenting gliomas are the Parvoviridae, Paramyxoviridae, Picornaviridae, Reoviridae, Retroviridae, Adenoviridae y Herpesviridae.

In this type of therapy, several obstacles must be overcome. The most important challenge is the lack of tropism of viruses through the brain, most are not able to cross the BBB, except Parvoviridae. Tumor resection causes neuroinflammation that can inactivate virus replication after surgery, so it is important to attend before virus administration. Moreover, the tumor microenvironment undergoes several modifications, including the development of extracellular matrix (ECM) associated with a desmoplastic state. Herpesviruses depend on this organization, especially its entry mediated by upregulated integrins in glioma. Blocking integrins would improve the viral replicative phenotype.

To prevent immune escape from glioma, several antigens are often used at once, rather than just a concrete antigen. In addition, it has been seen monotherapies with neoantigens do not achieve the maximum effect, but the use of classic combined therapies is convenient for attaining the maximum anti-tumor response. Despite all these efforts, treating glioma remains highly complex. This is not only due to the presence of an immunosuppressive environment but also because of the immune privilege environment, where the brain is located. Overcoming these challenges requires not only in the search for treatments that provoke an immune response but also the exploration of new forms of administration that are either able to cross the BBB or inoculated directly in the area of the tumor bypassing that brain barrier.

### Potential favorable factors to immunotherapy

There is increasingly compelling evidence that the long-term success of traditional chemotherapeutic agents and radiotherapy (RT) depends on immunological effects. Immunogenicity results from a combination of antigenicity and adjuvantness, and many anticancer drugs activate the adaptive stress response in malignant cells, thus promoting the emission of danger signals that function as immunological adjuvants.

RT and chemotherapy have both immunostimulatory and immunosuppressive effects. Clinical trial evaluation of immunotherapies in cancer patients have previously demonstrated that the combination of RT may be synergistic with immune checkpoint inhibitors across a wide range of advanced cancers [77]. It is thought RT promotes the data provide strong evidence that the H3.3K27M mutation is not a suitable target for cancer immunotherapy, most likely due to insufficient epitope processing and/or amount to be recognized by HLA-A*02:01 restricted CD8^+^ T cells [54]. On the other hand, in preclinical models, treatment with TMZ increases major histocompatibility complex 1 (MHC-I) expression on glioma cells through a nuclear factor of NF-κB dependent mechanism [143]. In other malignancies, there is clear synergy when traditional cytotoxic chemotherapies are combined with immunotherapies [35].

This data suggests that the careful immunological characterization of currently approved (and often relatively successful) anticancer agents may allow us to design ever more efficient and safe combinatorial regimens that build on existing therapeutic options.

### Implication of genetic alterations in glioma on immunotherapeutic efficacy

The mutation landscape of glioblastoma has been deeply characterized, which has allowed an easier determination of the role of GBM specific genomic alterations in the response to immunotherapeutic. The IDH mutations (IDHmut), commonly found in less aggressive gliomas, are potential targets for immunotherapies as a tumor-specific neoantigen, as we mentioned above. Moreover, D-2-HG, the oncometabolite induced by IDHmut, induced DNA hypermethylation in gliomas results in suppression of immune cell attraction and silencing of PD1 and PDL1 compared to IDH wild-type gliomas [133]. This result led to the attempt to combine IDH inhibitors with immune checkpoint inhibitors resulting in increased overall survival [11]. On the other hand, EGFR mutation and vIII mutation are frequent in GBM. Studies have revealed that EGFR plays a role in regulating immune microenvironment and immune response [15, 107]. Particularly, EGFR mutation decreased INFγ response [57], which activates the anti-tumor response and is required for response to any type of immunotherapy. PTEN is another GBM driver ocnogene whose mutation is associated with immunosuppressive mechanism during ICB treatment of GBM and appear to be enriched in the non-responders [144]. The H3.3K27M mutation is found in the vast majority of diffuse midline glioma and is not a suitable target for cancer immunotherapy, most likely due to insufficient epitope processing and/or amount to be recognized by HLA-A restricted CD8^+^ T cells [54].

## Recent studies illustrating the use of personalized neoantigens in immunotherapy

Only a small fraction of the mutations can induce spontaneous immune responses in the tumor-bearing host, which limits efficacy of immunotherapy to tumors with a high mutational load [83, 124]. Moreover, a large fraction of the mutations in human tumors is not shared between patients at meaningful frequencies and may, therefore, be considered patient specific. Based on this data, it is plausible that neoantigen-specific T cell reactivity forms the key to cancer immunotherapies success. Therefore, there has been the enthusiasm for the development of personalized approaches vaccines in the last years.

In recent years, this approach has been extended to human cancers. Among them, highlight the case of a 43-year-old woman with widely metastatic cholangiocarcinoma who had progressed through multiple chemotherapy regimens was enrolled in a TIL-based ACT protocol for patients with GI cancers (NCT01174121) [125]. Whole exome sequencing revealed 26 nonsynonymous mutations, which were tested to determine whether any of the processed and presented mutated antigens were recognized by TIL. A mutation in erbb2 interacting protein (ERBB2IP) was selected. After adoptive transfer of TIL, containing about 25% mutation-specific poly-functional Th1 cells, the patient achieved a decrease in target lesions with prolonged stabilization of disease. Upon disease progression, the patient was retreated with a > 95% pure population of mutation reactive Th1 cells and again experienced tumor regression.

In patients with melanoma, a vaccine that targets personal neoantigens has been tested [90]. To generate it, whole exome sequencing of DNA from matched normal and tumor cells from individual patients was performed and somatic mutations were identified. These were validated by RNA-seq in the tumor, and it was predicted which mutated peptides, which were more likely to bind to autologous HLA-A or HLA-B proteins of the patients. A vaccine that targets up to 20 predicted personal tumor neoantigens was generated. Of six vaccinated patients, four had no recurrence 25 months after vaccination, while two patients, with recurrent disease, were subsequently treated with anti-PD-1 (anti-programmed cell death-1) therapy, experiencing a complete tumor regression, with an expansion of the repertoire of neoantigen-specific T cells. These data provided a strong rationale for further development of this approach, not only alone, but also in combination with checkpoint blockade or other immunotherapies.

Regarding GBM, the Glioma Actively Personalized Vaccine Consortium (GAPVAC) has conducted a phase I trial GAPVAC-101, which has been integrated highly individualized vaccinations with tumor antigens for patients with newly diagnosed glioblastoma [50] (NCT02149225). Fifteen patients with glioblastomas positive for HLA were treated with a personalized vaccine (APVAC1). Based on mutations and analyses of the transcriptomes and immunopeptidomes of the individual tumors. Patients that received vaccinations presented a median overall survival of 29.0 months from arrival, and one of the patients even had an overall survival of > 38.9 months. The achievements of the current trial and those mentioned above certainly warrant further studies to understand how anti-tumor immunity can be leveraged to achieve clinical benefit for patients with glioblastoma.

## Tumor neoantigens: therapeutic potential

### Neoantigen classification

#### Based on tissue expression

Today three types of tumor antigens have the potential to elicit immune responses: tumor specific antigens (TSAs), tumor-associated antigens (TAAs), and cancer-germline/cancer testis antigens (CTAs).

TSAs are antigens that are not encoded in the normal host genome and represent abnormal proteins that arise because of somatic mutations (i.e., neoantigens). During cancer initiation and progression, tumor cells acquire protein-altering mutations that are responsible for this transformation [130]. Some of these alterations may result in the expression of mutant proteins that are perceived as foreign proteins by the immune system.

TAAs include proteins encoded in the normal genome and may be either normal differenced antigens or aberrantly expressed normal proteins. Overexpressed normal proteins that possess growth/survival promoting functions represent TAAs. This is because a threshold level of antigen is required for recognition by T cells. If tumor cells present an amount of peptide–HLA complexes that is above the threshold and if normal cells do not a specific antitumoral T cell response could occur. Along these lines, TAAs usually have lower T cell receptor (TCR) affinity compared with TSAs or foreign antigens [114]. Some examples of these TSAs is growth factor receptor ERBB2 (also known as HER2 and NEU) which is overexpressed in many epithelial tumors, including ovarian and breast carcinomas [32]. Posttranslational modifications of proteins such as phosphorylation may also lead to the formation of TAAs [21]. When compared to TSAs, TAAs display two advantageous features. First, they are more numerous [69]. Indeed, in a recent study of 23 ovarian cancers, 103 tumor antigens were identified of which only three were TSAs. Second, whereas TSAs are generally unique to individual patients, TAAs are shared by many tumors. In ovarian cancer, 78% of transcripts coding for individual TSAs were found in at least 10% of tumors and 18% in at least 80% of tumors [145].

The third category comprises CTAs, which not only are encoded by genes that are normally expressed in the human germline, but also expressed in various tumor types, including melanoma, and carcinomas of the bladder, lung, and liver. These immunogenic proteins are being vigorously pursued as targets for therapeutic cancer vaccines [110]. The mechanism that leads to the activation of these genes in tumor cells involves the demethylation of their promoter, which is methylated in all normal cells except in germline cells [25, 41]. This demethylation seems to be more frequent in advanced tumors, which concurs with the increasingly aberrant pattern of DNA methylation that occurs during tumor progression.

In the last decade, there has been an increased interest in the study of tumor-specific antigens as therapeutic targets for cancer immunotherapy, with most of the efforts focusing on identifying TAAs and CTAs. The initial efforts concentrated on discovering TSAs encoded by mutated genes using massively sequencing approaches comparing DNA isolated from tumor versus normal sources [110]. Since the genome is large (3 billion base pairs) and has a complex analysis, the new technology has allowed investigators to focus only on the 1% of the genome that comprises the coding exons of known genes (Exome analysis). Moreover, it is also interesting to note that recent technical innovations have reduced the time for this approach, being now feasible to generate exome capture data and produce a list of somatic mutations in about three days. Mutation calling from exome capture sequencing data is achieved by aligning sequence reads to reference genomes. Additionally, tumor variant calls are compared with data from a matched normal tissue DNA.

On the other hand, there are TAAs, antigens that are generated by non-protein coding (non-exonic sequences) or by epigenetic and splicing aberrations which lead to the appearance of numerous proteins that are not found in normal cells. These variants are difficult to identify, especially from exome-capture data. In all cases, the use of RNA data from cDNA capture sequencing (cDNA Cap-Seq) or RNA-Seq is necessary to identify and/or confirm these types of antigens. In the case of overexpression, epitope abundance is estimated by quantitating RNA expression levels through quantitative reverse transcription PCR of humoral and normal tissues along with Immunohistochemically analysis.

Finally, the mutations defined by tumor-to-normal DNA comparisons are subjected to bioinformatic analysis to predict their immunogenicity. Currently, the most useful epitope prediction algorithms are those focused on binding peptides to MHC class I (MHC-I) molecules. In both humans and mice, the MHC-I antigen presentation pathway is responsible for presenting peptides derived from endogenous cell-intrinsic proteins to CD8^+^ CTL [7]. Multiple tools to predict peptide binding to MHC-I exist. A subset of these algorithms predicts peptide binding to different MHC-I variants based on artificial neural networks, providing predicted IC50 as an output [79]. In this category, NetMHC [78] is one of the most commonly used and best-validated prediction programs.

With the combination of next generation sequencing, in silico epitope prediction, and immunological approaches, it has been possible to identify and validated distinct TSAs in different types of tumor cells, such as murine B16-F10 melanoma [13] or sarcoma [83].

In this regard, there is great enthusiasm for treating malignant brain tumors with cancer immunotherapies due to successes in other cancers. Therefore, there is a need for the identification and targeting of tumor-specific antigens in order to potently stimulate T cells against GBM. To this end, other groups have addressed antigen discovery in GBM preclinical model [59], because the response to checkpoint blockade immunotherapy is likely influenced by the presence of neoantigens [103]. Glioblastomas harbor fewer than 100 exome-wide mutations [119], with only a subset representing candidate neoantigens. However, a subset of hypermutated glioblastomas has been described in which mutational loads can be 10–50-fold higher than average [61], but this genotype is only observed in approximately 25% of recurrent glioblastomas following temozolomide treatment. Moreover, there is limited intratumorally infiltration of immune cells in GBMs [99], for this reason exploitation of the full repertoire of tumor antigens, that is, both unmutated antigens and neoepitopes, may offer a more effective immunotherapies, especially for tumors with low mutational load like gliomas.

Characterization of tumor-specific mutations expressed in GBM has been performed in three murine brain tumor models, GL261, SMA-560 and CT2A by exome sequencing followed by RNA sequencing [60, 74]. In these studies, the presence of 4932, 2171, and 2401 non-synonymous exome mutations, respectively, have been determined, of which less than half are expressed. In addition, candidate immunogenic antigens have been established in silico by predictive evaluation of the affinity of antigens to activate tumor-infiltrating T cells. Some top-ranking candidates were screened by neoantigens vaccination, as in the case of CT2A. Of the 29 CT2A neoantigens screened, it has been identified endogenous neoantigens-specific CD8^+^ T cells within an αPD-L1 resistant murine GBM. These observations show that neoantigen vaccination significantly augments survival benefit in combination with αPD-L1 treatment supporting further investigation studying the effects of multi-modal immunotherapeutic interventions on anti-glioma immunity.

#### Based on clinical setting

Guarding neoantigen. A group of neoantigen-specific T cells that can be activated before the clinical appearance of the tumor was described, characteristically, the presence of these neoantigens is enough to induce a relevant clinical effect in the absence of immunotherapy, i.e., they can help to accelerate or reject the tumor [68].Two types of guarding neoantigen are recognized: the immunodominant, caused by exceptionally rare mutations, which contribute to the improvement of clinical prognosis in tumors with a high mutational charge, such as, microsatellite instability. The second type is recognized by pre-established cross-reactive memory T cells, with have a lower activation threshold regarding memory cells. Those neoepitopes able to stimulate a more diverse TCR repertoire, such as those with a higher dissimilarity with self-antigens, will have more probabilities of belonging to this subclass of cross-reactive neoantigens [68]. Those with low affinity for MHC, low stability in peptide-MHC complex binding, or without enough neoantigen expression for per-forming naive T-cell priming in LM, may be compromised, and expanded by cross-reactive memory T cells.

Regarding treatment, ICB o neoantigen vaccine can increase the response of pre-existing T cells quantitatively or qualitatively against guarding neoantigen.

Restrained neoantigen. Not all T cells that occur spontaneously against a specific epitope are functional; they need further invigoration, such as ICB. Compared to guarding neoantigens, which are identified depending on their prognosis impact, the restrained are defined for their predictive capacity for the immuno-therapy clinical benefit [68].

Ignored neoantigens. Only a small fraction of neoantigens is recognized by spontaneous T cells, indeed, a big proportion of the generated immune responses were not detectable before the therapy and were induced after vaccination. Frankziska Lang et al*.* [68] proposed ignored neoantigens term for such cases. These neoantigens are present in MHC molecules, however, they need vaccination for achieve a relevant clinical response, in fact, the purpose of the vaccine is to induce antigen-specific LN resident DCs and achieve cell priming.

Interestingly, T cells induced by the vaccine increase PD1 levels, therefore, even those patients’ resistant to ICB monotherapy can benefit from the ICB and vaccine combining therapy. The key point is that vaccines can increase the number of preexisting T cells, increasing simultaneously the number of ignored neoantigens ready for ICB. Besides, when counteracting the immunosuppressive mechanism performed by T regulatory cells, the ICB can decrease the presentation threshold required for naive T cells priming, thereby expanding T cell response by antigen spreading.

### Identification of neoantigens

**Fig. 3:**
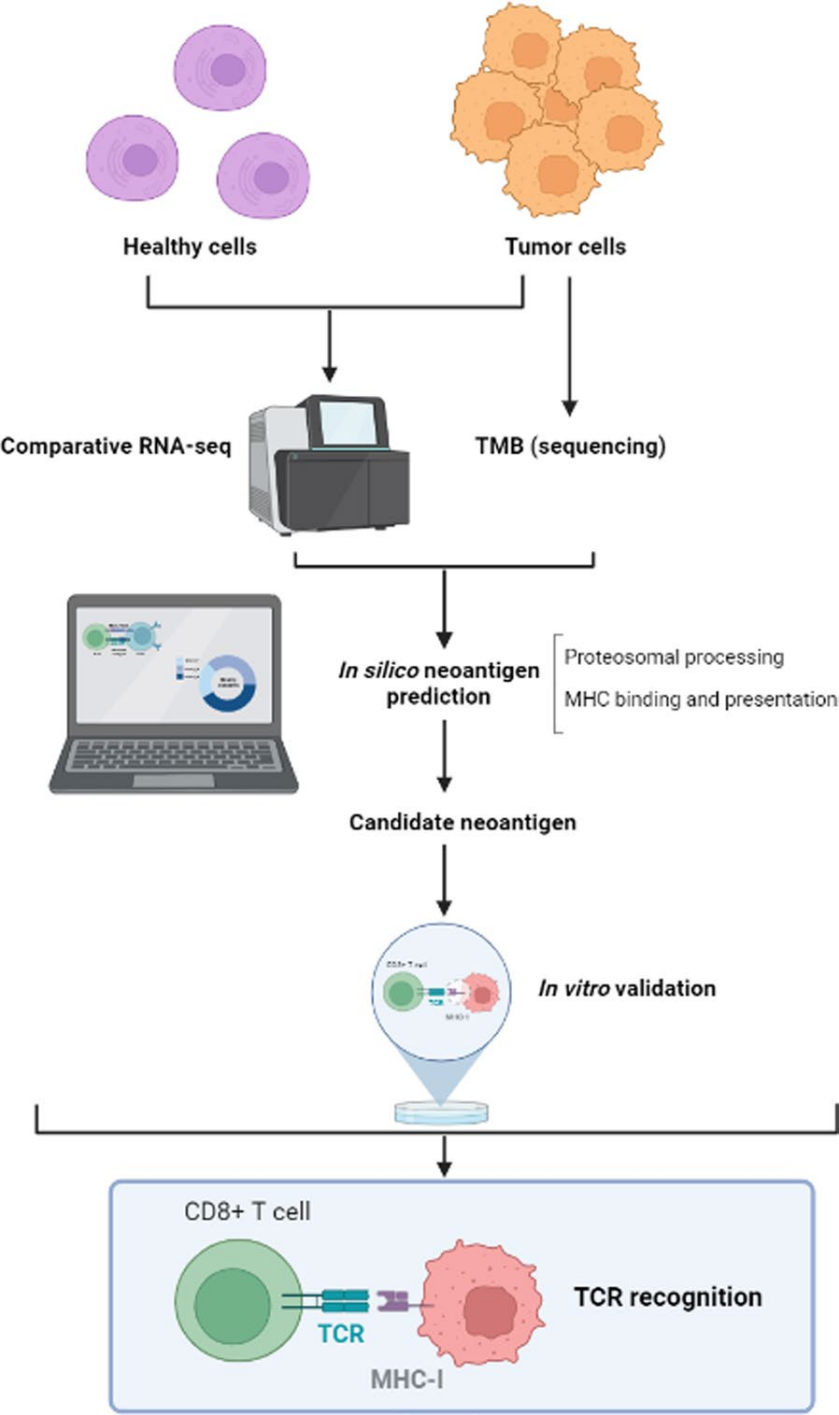
Representative scheme of possible neoantigens determination and validation procedure. Healthy and tumor cells comparative RNA sequencing will be performed, with the use of sophisticated software a complete analysis for neoantigen prediction will be done, continuing with an in vitro validation

As it was mentioned, computational approaches are being used to achieve a better characterization of the neoantigens, these advances permit us to attain better predictable neoantigen candidates (Fig. 3).

Immunobiology-driven approaches: In order to elicit an immune response is necessary to process the molecule and present it to MHC, therefore, two key factors in computational neoantigen prediction in the patient are: expression verification and prediction of the affinity binding of the MHC alleles. Asides from these factors, other biological characteristics are being applied in the algorithm, including those affecting the skillfulness of a presented neoepitope candidate to activate T cells [68]. Further studies are needed to correlate these characteristics with context-based neoantigen classes and establish how to analyze each of these neoantigens and prioritize the one´s candidates for vaccine design.

Transcript expression: Peptide-MHC complex density is correlated with the protein levels and transcript expression. Those neoantigens expressed by tumoral cell clones with a high transcript abundance are efficiently eliminated under ICB therapy, whereas down expression of the neoantigen candidates is used an immune escape strategy.

To summarize, the high expression of the transcript is associated with a higher probability of functional spontaneous response of T cells.

MHC binding, stability, and cell surface presentation: The ability of a mutant peptide to bind to at least one of the patient's MHC alleles is a key requirement for T-cell recognition. The collaboration of antigen specific CD4^+^ and CD8^+^ T cells is crucial for effective antitumor immunity. Expression of a single MHC-I neoantigen is not sufficient, and at least one additional MHC-II neoantigen is required for significant antitumor immunity in mouse models with tumors [68]. Consequently, the individualized vaccine should combine neoepitopes that are predicted to bind MHC I and II alleles. Moreover, it has been proposed that the stability of the MHC-peptide complex is more important than the binding affinity for predicting immunogenicity due to a higher probability of T cell recognition [68].

Dissimilarity to self and similarity to pathogen-associated: The more the neoepitope resembles pathogenic sequences, the greater the likelihood of cross-reactivity with preformed T cells against frequent pathogens.

*TCR recognition* TCR-peptide-MHC complex interactions are based on predictions of TCR amino acids chains that will participate in MHC-peptide binding or the stability of the complex. This is associated with an increased probability of TCR binding.

Mutation clonality and indispensability: Clonal and truncal mutations are preferable to subclonal and branching mutations because they address tumor heterogeneity, and focus on tumorigenic mutations with higher fitness and tumor-promoting functions [68].

*LOH* LOH may be a good target for vaccines, if an important gene undergoes LOH and presents a neoantigen, the tumor cannot escape due to loss of antigen since the remaining allele is necessary for tumor cell survival.

*Deep learning-based approaches* It is used for immunogenicity prediction, however, the lack of a sufficiently long and standardized data set with high-quality T cell response data and the difficulty in discrimination between data sets reflecting immunogenicity versus those showing antigenicity is a major obstacle for using this tool [68].

As in any therapeutic strategy, there are also find a series of obstacles in using neoantigen-based vaccines, with most being technological challenges. First, we have the challenge of using bio-samples as analytes. A cancer-specific biomarker exhibits a wide heterogeneity, and multiple biopsies from the same tumoral lesion have different molecular profiles. Furthermore, the candidate neoantigens identified in one patient's metastatic lesion differ from those present in a second metastasis or primary tumor. Secondly, in mutation calling, it is important to distinguish that the results are not a consequence of sequencing errors, sample preparation artefacts, or germline mutations. Thirdly, it is important to establish the parameters for neoantigen prediction algorithms, which depends on the availability of well-preserved data sets [68]. The defiance is the lack of harmonized protocols for sequencing, mutation detection, prioritization of neoantigen candidates and immunogenicity testing for data integration and comparability.

## Customized tumoral vaccines

Cancer immunotherapy has shown great potential by saving the lives of a proportion of late-stage patients with immunogenic tumor types. However, even in these sensitive tumor types, most patients still do not sufficiently respond to the therapy. Furthermore, other tumor types, including gliomas, remain largely refractory. Gliomas harbor a lower burden of somatic mutations, fewer infiltrative T cells and an immunosuppressive TME [94].

Although the importance of the TME is well established, comprehensive analyses based on its TME remain lacking. Clearly understanding the cancer type- and treatment response-specific variations in the TME may elucidate the mechanisms underlying therapeutic resistance. In this sense, significant effort has been made to characterize the principal process that affects the response of immunotherapy: the characterization of the immunosuppressive TME, the antigenic presentation and the presence of tumor-infiltrating lymphocytes. These investigations have provided important insights into understanding the complexity of TME, even stratifying tumors into TME subtypes. In one of them, tumors are categorized into the following three groups based on inflamed: Inflamed tumors which are characterized by the presence of tumor-infiltrating lymphocytes, high density of IFNγ-produced by T cells and high expression of PD-L1 [48]. In contrast, non-inflamed tumors are poorly infiltrated by lymphocytes, rarely express PD-L1, and are characterized by low expression of antigen presentation machinery markers including MHC-I [48]. In between these two groups are the excluded tumors, with infiltration of immune cells but are excluded from the tumor, with peritumoral T-cell localization [48].

On the other hand, Thorsson et al*.* performed an extensive immunogenomic analysis of 33 different types of cancers utilizing data from the cancer genome atlas (TCGA). With this study they were able to establish six immune subtypes: wound healing, IFN-γ dominant, inflammatory, lymphocyte depleted, immunologically quiet, and TGF-β dominant, based on the extent of neoantigen load, differences in macrophage and lymphocyte signatures, Th1/Th2 cell ratio, expression of immunomodulatory genes, and prognosis, among others [120].

Another study in melanoma TME also using TCGA data, has clustered the cohort in four distinct microenvironments, based on different gene expression signatures of the major cell components (immune, vascular, and stromal populations) which are called Immune-enriched/fibrotic (IE/F), immune-enriched/non-fibrotic (IE), fibrotic (F) and immune-depleted (D) [4]. The IE/F subtype was characterized by elevated angiogenesis and CAF activation. The IE melanomas were distinguished from IE/F subtype by high levels of immune infiltrate and a more immune-active microenvironment. IE melanomas also had the highest ratios of CD8^+^ T cells/Tregs and M1/M2 macrophages. The F and D subtypes possessed minimal leukocyte/lymphocyte infiltration, with subtype D containing the highest malignant cell percentage. Melanomas with F subtypes showed elevated angiogenesis and increased CAFs, which are strong immune suppressors and TME remodelers via secretion of TGF-β [16]. In addition, they also established that this expression signature-based TME classification system can be broadly applied at the pan-cancer level, but the significance of this TME subtyping, needed to be further evaluated within individual cancer types. Moreover, they have also been able to establish how TME directly influences the effectiveness of the immune checkpoint blockade. Tumors from melanoma patients, who responded to immunotherapy evolved to IE and IE/F subtypes, namely the immune-enriched TME. Whereas the non-responders retained the immune unfavorable subtype F.

The observed TME subtypes in different studies share multiple similarities with the clusters identified in all of them, reflecting or expanding upon the same patterns. In this way, three common TME in all cancers can be established: Inflamed, excluded and desertic TIME.

**Fig. 4:**
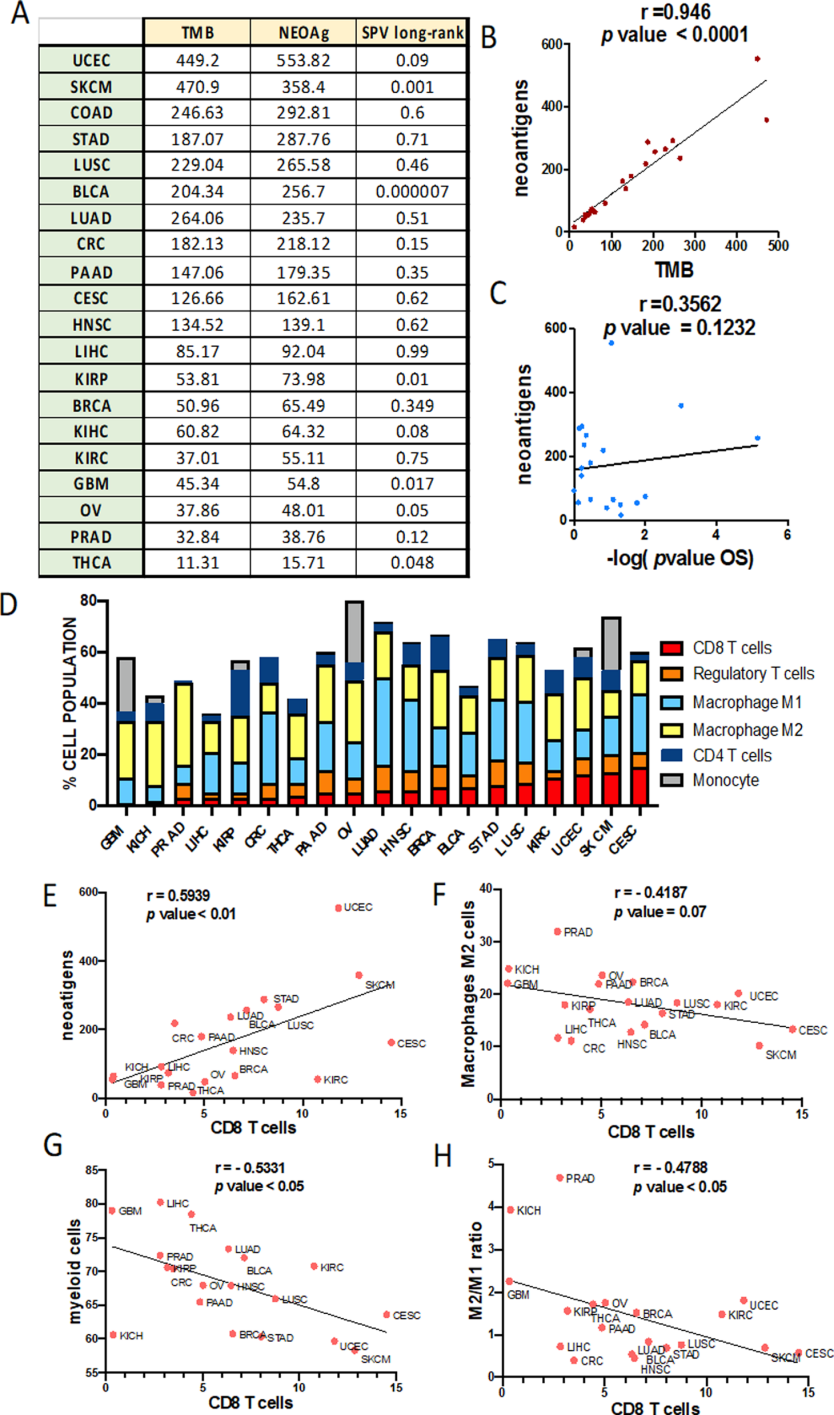
Tumor neoantigens and cellular Characterization of Immune Infiltrates in Solid Cancers. **A** Tumor mutational burden (TMB), neoantigen load and Long-rank survival analysis based on a high and low amount of neoantigens in each tumor from TCGA cohort. **B**, **C** Correlation between neoantigen load and the TBM (**B**) or *p* value of survival analysis (**C**) in each tumor. **D** Percentage of CD8 positive T cells, regulatory T cells, Macrophague M1, Macrophague M2, CD4 positive T cells and monocyte populations on total CD45 suspension in each type of tumor from the TCGA cohort. **E** Correlation between neoantigen load and CD8 positive T cells in each tumor. **F**–**H**, Correlation between CD8 positive T cells and myeloid cells (**F**), macrophages M2 cells (**G**) and the ratio of M2/M1 cells (**H**) in each tumor from TCGA cohort

Tumor antigens with the potential to elicit immune responses, which are strictly tumor-specific, are the neoantigens that result from mutations. The neoantigen landscape in solid tumors (Pan-Cancer cohort) was composed of 933,954 expressed neoantigens (data retrieved from http://tcia.at) and as we expected, the number of neoantigens correlated with the mutational load across all cancers (Fig. 4A–B). For this reason, the TMB has allowed the establishment of tumors groups with a possible sensitivity to ICI [36].

On the other hand, we observed that neoantigens do not show a positive relationship with overall survival (Fig. 4C). In addition, the immune cell subpopulations are highly variable between different cancer entities (Fig. 4D), which results suggests that the immune response is likely governed by combined effects. The differences in immunogenicity of the tumors are determined by TMB and infiltration of TILs, which is the parameter used to define “cold” or “hot” tumors [36]. Accordingly, we can observe how, cancers with poor response to ICI such as prostate cancer (PRAD) or GBM, have a low percentage of TILs (CD8 positive cells). Nevertheless, melanoma (SKCM) or kidney renal clear cell carcinoma (KIRC), which are ICI-responsive tumors, show a high content of CD8 positive cells (Fig. 4D). Somewhat unexpected, there is a positive correlation between the number of neoantigens and the infiltrate of T cells (Fig. 4E) while there is an inverse correlation between T cells and myeloid cells (Fig. 4F), and specifically with the immunosuppressive phenotype (M2) of this type of cells (Fig. 4G–H). These analyses showed that the content of M2 macrophage is the most common cell type significantly associated with the suppressor of immune response activation in all cancers.

In summary, there are certain tumors or subtypes of specific tumors that have disadvantageous microenvironments for immune activation. The stratifications mentioned above reflect clear evidence that vaccines need specific stimuli in order to initiate an immune response in this unfavorable TMEs, in case of GBM. Thus, there are a large number of ongoing studies focused on regulating innate immune response, which make it possible to reduce suppressor myeloid populations such as M2 macrophages or MDSCs,, in order to favor DCs function and antigen processing [82]. Specifically, in the case of GBM, it has been observed that CD39 and CD73 are key in the regulation of the TME [116], generating strong immune activation effects [39]. In this sense, macrophages can be reprogrammed using vascular remodelers such as dual anti-Tie2 Activation and Ang2 inhibitor [92]. This can be achieved using Ang-2/VEGFA bispecific antibody [64] or combining bevacizumab and autophagy activators [19].

There are also myeloid recruitment inhibitors such as CCR2 [33] or CSFR1 inhibitors [97]. In addition, more specifically, the antigenic presentation can be improved with myeloid cell-specific phagocytosis checkpoint, like antibodies against SIRPα-CD47 [38, 53, 58, 131].

## Discussion (conclusion and future perspective)

Because only a minority of patients are responsive to checkpoint blockers, major efforts are underway to understand the mechanism behind this and develop therapeutic strategies to overcome resistance While T cell-activating immunotherapies such as anti-PD1 have led to remarkable responses in brain metastases [118], outcomes in the treatment of GBM have been disappointing due to the immunosuppressive TME [104].

Currently, high-precision genomic techniques have allowed us to understand the complexity of the immune microenvironment, leading to clustering of nearly all human tumor types in six immune subtypes by a meta-analysis of consensus expression [120]. These subtypes were associated with prognosis, genetic, and immune-modulatory alterations that shape the specific types of immune TME and response to therapy. The glioma belongs to the subtype with lymphocyte depletion, accompanied by prominent macrophage signature, Th1 suppressed and a high M2 response.

In this sense, it is known that the number of cytotoxic CD8 T cells is critically important to mediate the effects of immunotherapy. For this reason, the paucity of CD8 T cells in GBM has long been considered the reason for the failure of immunotherapies. On the contrary, in the last few years, different studies carried out by others [62] have indicated that CD8 T cells have a great influence on the immunogenicity and the microenvironment through immunoediting, but these cells might not be the unique responsible for ICI response. This may be related to an unappreciated role of the innate immune system in modulating malignant degeneration [34]. The population of PD1 + macrophages is very robust in GBM and may be targeted by ICI more than T cells, due to the oncologic setting characterized by the absence of these. It has been observed that ICI targeting PD1 results in significant survival gains in immunocompetent mice even when CD8 T cells are absent [101]. Treatment with anti–PD1 antibody shifts the polarization of remaining macrophages to antitumor M1 phenotype. In previous clinical trials of GBM, patients [20, 24] were treated with anti–PD1 prior to surgery. Immune profiling of the tumor microenvironment revealed a marked paucity of effector T cells but a profound predominance of macrophages immune-stimulatory and immune-suppressive phenotypes. It is likely that he therapeutic effect of anti-PD1 can shift between various immune populations, depending on their relative frequencies. In malignancies enriched in T-cell infiltration, anti-PD1 likely exerts most of its therapeutic activity through direct T-cell interactions. In contrast, in malignancies such as glioblastoma that are devoid of T cells, anti-PD1 activity may exert a therapeutic effect through alternative immune populations such as macrophages and microglia. This alternative mechanism provides an explanation for the failure of immunotherapies in cancers such as GBM, where the focus is mainly on the immune functional features of the adaptive immune system such as the abundance of antigens, the presence of T-cell infiltration and ligand frequency. Leaving aside the innate response, which as our data also shows (Fig. 4), it could be the key to improving immunotherapy in non-responsive cancers.

In agreement with the idea that not only T cells have the strongest involvement in the response to immunotherapy, it has been possible to establish the dynamics in the functioning and activation of the immune system in preclinical models, defining a cancer-immunity cycle [47]. It is comprised of seven steps including the release of cancer antigens, antigen presentation, immune activation, trafficking, infiltration, specific recognition of cancer cells by T cells, and killing of cancer cells. Developing the optimal treatment may require multiple therapeutics to modulate each step required to generate an effective immune response to cancer.

To date, it is well known that cellular vaccines can be considered a promising therapeutic strategy for glioma patients. Among all the therapies that have demonstrated significant benefit for gliomas in clinical trials, including radiation, chemotherapy (TMZ and PCV) and targeted therapies (bevacizumab) [122], the impact of cellular vaccine therapies has been the most modest in glioma, in addition to demonstrating that is a therapy feasible and generally well tolerated. On the other hand, combining cellular vaccine with immune response modifiers in glioma promises to boost the true power of cellular vaccines and potentially offer long-term protection from tumor recurrence, although there is a substantial debate about the optimal combination of immunotherapeutic modalities. Some of the most used immunomodulatory in brain tumors in combination with vaccines are Poly-ICLC, which is utilized with a diverse number of neoantigen vaccines, most commonly in peptide and mRNA vaccines (clinical trial examples: NCT03068832, NCT02287428). It is a product which stimulates innate immunity by promoting pattern recognition receptors, TLR3 and MDA5. These receptors stimulate the activation of cytokines IFN-I and IL-15, enhancing T-cell responses and promoting T-cell expansion [115]. GM-CSF is a proinflammatory factor commonly used with peptide and DNA vaccines, which enhances T-cell activation and the function of DCs [146].

In summary, while many unknowns, questions, and challenges with vaccine-based immunotherapy still inevitably remain, most agree that we are off to a new age in cancer immunotherapy.

## References

[1] Alexandrov LB, Nik-Zainal S, Wedge DC, Aparicio SA, Behjati S, Biankin AV, Bignell GR, Bolli N, Borg A, Børresen-Dale AL (2013). Signatures of mutational processes in human cancer. Nature.

[2] Ardon H, Van Gool SW, Verschuere T, Maes W, Fieuws S, Sciot R, Wilms G, Demaerel P, Goffin J, Van Calenbergh F (2012). Integration of autologous dendritic cell-based immunotherapy in the standard of care treatment for patients with newly diagnosed glioblastoma: results of the HGG-2006 phase I/II trial. Cancer Immunol Immunother.

[3] Arvanitis CD, Ferraro GB, Jain RK (2020). The blood-brain barrier and blood-tumour barrier in brain tumours and metastases. Nat Rev Cancer.

[4] Bagaev A, Kotlov N, Nomie K, Svekolkin V, Gafurov A, Isaeva O, Osokin N, Kozlov I, Frenkel F, Gancharova O (2021). Conserved pan-cancer microenvironment subtypes predict response to immunotherapy. Cancer Cell.

[5] Berghoff AS, Kiesel B, Widhalm G, Rajky O, Ricken G, Wöhrer A, Dieckmann K, Filipits M, Brandstetter A, Weller M (2015). Programmed death ligand 1 expression and tumor-infiltrating lymphocytes in glioblastoma. Neuro Oncol.

[6] Bloch O, Lim M, Sughrue ME, Komotar RJ, Abrahams JM, O'Rourke DM, D'Ambrosio A, Bruce JN, Parsa AT (2017). Autologous heat shock protein peptide vaccination for newly diagnosed glioblastoma: impact of peripheral PD-L1 expression on response to therapy. Clin Cancer Res.

[7] Blum JS, Wearsch PA, Cresswell P (2013). Pathways of antigen processing. Annu Rev Immunol.

[8] Boussiotis VA, Charest A (2018). Immunotherapies for malignant glioma. Oncogene.

[9] Bowman RL, Joyce JA (2014). Therapeutic targeting of tumor-associated macrophages and microglia in glioblastoma. Immunotherapy.

[10] Brown CE, Alizadeh D, Starr R, Weng L, Wagner JR, Naranjo A, Ostberg JR, Blanchard MS, Kilpatrick J, Simpson J (2016). Regression of glioblastoma after chimeric antigen receptor T-cell therapy. N Engl J Med.

[11] Bunse L, Pusch S, Bunse T, Sahm F, Sanghvi K, Friedrich M, Alansary D, Sonner JK, Green E, Deumelandt K (2018). Suppression of antitumor T cell immunity by the oncometabolite (R)-2-hydroxyglutarate. Nat Med.

[12] Burger MC, Zhang C, Harter PN, Romanski A, Strassheimer F, Senft C, Tonn T, Steinbach JP, Wels WS (2019). CAR-engineered NK cells for the treatment of glioblastoma: turning innate effectors into precision tools for cancer immunotherapy. Front Immunol.

[13] Castle JC, Kreiter S, Diekmann J, Löwer M, van de Roemer N, de Graaf J, Selmi A, Diken M, Boegel S, Paret C (2012). Exploiting the mutanome for tumor vaccination. Cancer Res.

[14] Castro Dias M, Mapunda JA, Vladymyrov M, Engelhardt B (2019). Structure and junctional complexes of endothelial, epithelial and glial brain barriers. Int J Mol Sci.

[15] Cejalvo T, Gargini R, Segura-Collar B, Mata-Martínez P, Herranz B, Cantero D, Ruano Y, García-Pérez D, Pérez-Núñez Á, Ramos A (2020). Immune profiling of gliomas reveals a connection with IDH1/2 mutations tau function and the vascular phenotype. Cancers (Basel).

[16] Chakravarthy A, Khan L, Bensler NP, Bose P, De Carvalho DD (2018). TGF-β-associated extracellular matrix genes link cancer-associated fibroblasts to immune evasion and immunotherapy failure. Nat Commun.

[17] Charles N, Holland EC (2010). The perivascular niche microenvironment in brain tumor progression. Cell Cycle.

[18] Chen RQ, Liu F, Qiu XY, Chen XQ (2018). The prognostic and therapeutic value of PD-L1 in glioma. Front Pharmacol.

[19] Chryplewicz A, Scotton J, Tichet M, Zomer A, Shchors K, Joyce JA, Homicsko K, Hanahan D (2022). Cancer cell autophagy, reprogrammed macrophages, and remodeled vasculature in glioblastoma triggers tumor immunity. Cancer Cell.

[20] Cloughesy TF, Mochizuki AY, Orpilla JR, Hugo W, Lee AH, Davidson TB, Wang AC, Ellingson BM, Rytlewski JA, Sanders CM (2019). Neoadjuvant anti-PD-1 immunotherapy promotes a survival benefit with intratumoral and systemic immune responses in recurrent glioblastoma. NatMed.

[21] Cobbold M, De La Peña H, Norris A, Polefrone JM, Qian J, English AM, Cummings KL, Penny S, Turner JE, Cottine J (2013). MHC class I-associated phosphopeptides are the targets of memory-like immunity in leukemia. Sci Transl Med.

[22] Coniglio SJ, Segall JE (2013). Review: molecular mechanism of microglia stimulated glioblastoma invasion. Matrix Biol.

[23] Constam DB, Philipp J, Malipiero UV, ten Dijke P, Schachner M, Fontana A (1992). Differential expression of transforming growth factor-beta 1, -beta 2, and -beta 3 by glioblastoma cells, astrocytes, and microglia. J Immunol.

[24] de Groot J, Penas-Prado M, Alfaro-Munoz K, Hunter K, Pei BL, O'Brien B, Weathers SP, Loghin M, Kamiya Matsouka C, Yung WKA (2020). Window-of-opportunity clinical trial of pembrolizumab in patients with recurrent glioblastoma reveals predominance of immune-suppressive macrophages. Neuro Oncol.

[25] De Smet C, De Backer O, Faraoni I, Lurquin C, Brasseur F, Boon T (1996). The activation of human gene MAGE-1 in tumor cells is correlated with genome-wide demethylation. Proc Natl Acad Sci U S A.

[26] Di Nunno V, Franceschi E, Gatto L, Bartolini S, Brandes AA (2020). Predictive markers of immune response in glioblastoma: hopes and facts. Future Oncol.

[27] Draaisma K, Wijnenga MM, Weenink B, Gao Y, Smid M, Robe P, van den Bent MJ, French PJ (2015). PI3 kinase mutations and mutational load as poor prognostic markers in diffuse glioma patients. Acta Neuropathol Commun.

[28] Eagles ME, Nassiri F, Badhiwala JH, Suppiah S, Almenawer SA, Zadeh G, Aldape KD (2018). Dendritic cell vaccines for high-grade gliomas. Ther Clin Risk Manag.

[29] Emami Nejad A, Najafgholian S, Rostami A, Sistani A, Shojaeifar S, Esparvarinha M, Nedaeinia R, Haghjooy Javanmard S, Taherian M, Ahmadlou M (2021). The role of hypoxia in the tumor microenvironment and development of cancer stem cell: a novel approach to developing treatment. Cancer Cell Int.

[30] Engelhardt B, Vajkoczy P, Weller RO (2017). The movers and shapers in immune privilege of the CNS. Nat Immunol.

[31] Filley AC, Henriquez M, Dey M (2017). Recurrent glioma clinical trial, CheckMate-143: the game is not over yet. Oncotarget.

[32] Fisk B, Blevins TL, Wharton JT, Ioannides CG (1995). Identification of an immunodominant peptide of HER-2/neu protooncogene recognized by ovarian tumor-specific cytotoxic T lymphocyte lines. J Exp Med.

[33] Flores-Toro JA, Luo D, Gopinath A, Sarkisian MR, Campbell JJ, Charo IF, Singh R, Schall TJ, Datta M, Jain RK (2020). CCR2 inhibition reduces tumor myeloid cells and unmasks a checkpoint inhibitor effect to slow progression of resistant murine gliomas. Proc Natl Acad Sci USA.

[34] Gajewski TF, Schreiber H, Fu YX (2013). Innate and adaptive immune cells in the tumor microenvironment. Nat Immunol.

[35] Galluzzi L, Buqué A, Kepp O, Zitvogel L, Kroemer G (2015). Immunological effects of conventional chemotherapy and targeted anticancer agents. Cancer Cell.

[36] Galon J, Bruni D (2019). Approaches to treat immune hot, altered and cold tumours with combination immunotherapies. Nat Rev Drug Discov.

[37] Gargini R, Segura-Collar B, Sánchez-Gómez P (2020). Cellular plasticity and tumor microenvironment in gliomas: the struggle to hit a moving target. Cancers (Basel).

[38] Gholamin S, Mitra SS, Feroze AH, Liu J, Kahn SA, Zhang M, Esparza R, Richard C, Ramaswamy V, Remke M (2017). Disrupting the CD47-SIRPα anti-phagocytic axis by a humanized anti-CD47 antibody is an efficacious treatment for malignant pediatric brain tumors. Sci Transl Med.

[39] Goswami S, Walle T, Cornish AE, Basu S, Anandhan S, Fernandez I, Vence L, Blando J, Zhao H, Yadav SS (2020). Immune profiling of human tumors identifies CD73 as a combinatorial target in glioblastoma. Nat Med.

[40] Gromeier M, Brown MC, Zhang G, Lin X, Chen Y, Wei Z, Beaubier N, Yan H, He Y, Desjardins A (2021). Very low mutation burden is a feature of inflamed recurrent glioblastomas responsive to cancer immunotherapy. Nat Commun.

[41] Grunau C, Sanchez C, Ehrlich M, van der Bruggen P, Hindermann W, Rodriguez C, Krieger S, Dubeau L, Fiala E, De Sario A (2005). Frequent DNA hypomethylation of human juxtacentromeric BAGE loci in cancer. Genes Chromosomes Cancer.

[42] Hambardzumyan D, Bergers G (2015). Glioblastoma: defining tumor niches. Trends Cancer.

[43] Hambardzumyan D, Gutmann DH, Kettenmann H (2016). The role of microglia and macrophages in glioma maintenance and progression. NatNeurosci.

[44] Han J, Chu J, Keung Chan W, Zhang J, Wang Y, Cohen JB, Victor A, Meisen WH, Kim SH, Grandi P (2015). CAR-engineered NK cells targeting wild-type EGFR and EGFRvIII enhance killing of glioblastoma and patient-derived glioblastoma stem cells. Sci Rep.

[45] Han J, Hong Y, Lee YS (2017). PD-L1 expression and combined status of PD-L1/PD-1-positive tumor infiltrating mononuclear cell density predict prognosis in glioblastoma patients. J Pathol Transl Med.

[46] Hanahan D, Weinberg RA (2011). Hallmarks of cancer: the next generation. Cell.

[47] Hegde PS, Chen DS (2020). Top 10 challenges in cancer immunotherapy. Immunity.

[48] Hegde PS, Karanikas V, Evers S (2016). The where, the when, and the how of immune monitoring for cancer immunotherapies in the era of checkpoint inhibition. Clin Cancer Res.

[49] Henrik Heiland D, Ravi VM, Behringer SP, Frenking JH, Wurm J, Joseph K, Garrelfs NWC, Strähle J, Heynckes S, Grauvogel J (2019). Tumor-associated reactive astrocytes aid the evolution of immunosuppressive environment in glioblastoma. Nat Commun.

[50] Hilf N, Kuttruff-Coqui S, Frenzel K, Bukur V, Stevanović S, Gouttefangeas C, Platten M, Tabatabai G, Dutoit V, van der Burg SH (2019). Actively personalized vaccination trial for newly diagnosed glioblastoma. Nature.

[51] Hodges TR, Ott M, Xiu J, Gatalica Z, Swensen J, Zhou S, Huse JT, de Groot J, Li S, Overwijk WW (2017). Mutational burden, immune checkpoint expression, and mismatch repair in glioma: implications for immune checkpoint immunotherapy. Neuro Oncol.

[52] Huettner C, Paulus W, Roggendorf W (1995). Messenger RNA expression of the immunosuppressive cytokine IL-10 in human gliomas. Am J Pathol.

[53] Hutter G, Theruvath J, Graef CM, Zhang M, Schoen MK, Manz EM, Bennett ML, Olson A, Azad TD, Sinha R (2019). Microglia are effector cells of CD47-SIRPα antiphagocytic axis disruption against glioblastoma. Proc Natl Acad Sci U S A.

[54] Immisch L, Papafotiou G, Popp O, Mertins P, Blankenstein T, Willimsky G (2022). H3.3K27M mutation is not a suitable target for immunotherapy in HLA-A2. J Immunother Cancer.

[55] Jan CI, Tsai WC, Harn HJ, Shyu WC, Liu MC, Lu HM, Chiu SC, Cho DY (2018). Predictors of response to autologous dendritic cell therapy in glioblastoma multiforme. Front Immunol.

[56] Jardim DL, Goodman A, de Melo GD, Kurzrock R (2021). The challenges of tumor mutational burden as an immunotherapy biomarker. Cancer Cell.

[57] Ji H, Ba Y, Ma S, Hou K, Mi S, Gao X, Jin J, Gong Q, Liu T, Wang F (2021). Construction of interferon-gamma-related gene signature to characterize the immune-inflamed phenotype of glioblastoma and predict prognosis, efficacy of immunotherapy and radiotherapy. Front Immunol.

[58] Jiang N, Xie B, Xiao W, Fan M, Xu S, Duan Y, Hamsafar Y, Evans AC, Huang J, Zhou W (2022). Fatty acid oxidation fuels glioblastoma radioresistance with CD47-mediated immune evasion. Nat Commun.

[59] Johanns TM, Miller CA, Dorward IG, Tsien C, Chang E, Perry A, Uppaluri R, Ferguson C, Schmidt RE, Dahiya S (2016). Immunogenomics of hypermutated glioblastoma: a patient with germline POLE deficiency treated with checkpoint blockade immunotherapy. Cancer Discov.

[60] Johanns TM, Ward JP, Miller CA, Wilson C, Kobayashi DK, Bender D, Fu Y, Alexandrov A, Mardis ER, Artyomov MN (2016). Endogenous neoantigen-specific CD8 T cells identified in two glioblastoma models using a cancer immunogenomics approach. Cancer Immunol Res.

[61] Johnson BE, Mazor T, Hong C, Barnes M, Aihara K, McLean CY, Fouse SD, Yamamoto S, Ueda H, Tatsuno K (2014). Mutational analysis reveals the origin and therapy-driven evolution of recurrent glioma. Science.

[62] Kane JR, Zhao J, Tsujiuchi T, Laffleur B, Arrieta VA, Mahajan A, Rao G, Mela A, Dmello C, Chen L (2020). CD8. Clin Cancer Res.

[63] Keir ME, Butte MJ, Freeman GJ, Sharpe AH (2008). PD-1 and its ligands in tolerance and immunity. Annu Rev Immunol.

[64] Kloepper J, Riedemann L, Amoozgar Z, Seano G, Susek K, Yu V, Dalvie N, Amelung RL, Datta M, Song JW (2016). Ang-2/VEGF bispecific antibody reprograms macrophages and resident microglia to anti-tumor phenotype and prolongs glioblastoma survival. Proc Natl Acad Sci USA.

[65] Kmiecik J, Poli A, Brons NH, Waha A, Eide GE, Enger P, Zimmer J, Chekenya M (2013). Elevated CD3+ and CD8+ tumor-infiltrating immune cells correlate with prolonged survival in glioblastoma patients despite integrated immunosuppressive mechanisms in the tumor microenvironment and at the systemic level. J Neuroimmunol.

[66] Knudsen AM, Rudkjøbing SJ, Sørensen MD, Dahlrot RH, Kristensen BW (2021). Expression and prognostic value of the immune checkpoints galectin-9 and PD-L1 in glioblastomas. J Neuropathol Exp Neurol.

[67] Landry AP, Balas M, Alli S, Spears J, Zador Z (2020). Distinct regional ontogeny and activation of tumor associated macrophages in human glioblastoma. Sci Rep.

[68] Lang F, Schrörs B, Löwer M, Türeci Ö, Sahin U (2022). Identification of neoantigens for individualized therapeutic cancer vaccines. Nat Rev Drug Discov.

[69] Laumont CM, Vincent K, Hesnard L, Audemard É, Bonneil É, Laverdure JP, Gendron P, Courcelles M, Hardy MP, Côté C (2018). Noncoding regions are the main source of targetable tumor-specific antigens. Sci Transl Med.

[70] Lee KS, Lee K, Yun S, Moon S, Park Y, Han JH, Kim CY, Lee HS, Choe G (2018). Prognostic relevance of programmed cell death ligand 1 expression in glioblastoma. J Neurooncol.

[71] Li X, Wang B, Gu L, Zhang J, Gao L, Ma C, Liang X (2018). Tim-3 expression predicts the abnormal innate immune status and poor prognosis of glioma patients. Clin Chim Acta.

[72] Liau LM, Ashkan K, Tran DD, Campian JL, Trusheim JE, Cobbs CS, Heth JA, Salacz M, Taylor S, D'Andre SD (2018). First results on survival from a large phase 3 clinical trial of an autologous dendritic cell vaccine in newly diagnosed glioblastoma. J Transl Med.

[73] Liu C, Sage JC, Miller MR, Verhaak RG, Hippenmeyer S, Vogel H, Foreman O, Bronson RT, Nishiyama A, Luo L (2011). Mosaic analysis with double markers reveals tumor cell of origin in glioma. Cell.

[74] Liu CJ, Schaettler M, Blaha DT, Bowman-Kirigin JA, Kobayashi DK, Livingstone AJ, Bender D, Miller CA, Kranz DM, Johanns TM (2020). Treatment of an aggressive orthotopic murine glioblastoma model with combination checkpoint blockade and a multivalent neoantigen vaccine. Neuro Oncol.

[75] Louis DN, Perry A, Reifenberger G, Von DA, Figarella-Branger D, Cavenee WK, Ohgaki H, Wiestler OD, Kleihues P, Ellison DW (2016). The 2016 World Health Organization classification of tumors of the central nervous system: a summary. Acta Neuropathol.

[76] Louveau A, Smirnov I, Keyes TJ, Eccles JD, Rouhani SJ, Peske JD, Derecki NC, Castle D, Mandell JW, Lee KS (2015). Structural and functional features of central nervous system lymphatic vessels. Nature.

[77] Luke JJ, Lemons JM, Karrison TG, Pitroda SP, Melotek JM, Zha Y, Al-Hallaq HA, Arina A, Khodarev NN, Janisch L (2018). Safety and clinical activity of pembrolizumab and multisite stereotactic body radiotherapy in patients with advanced solid tumors. J Clin Oncol.

[78] Lundegaard C, Lamberth K, Harndahl M, Buus S, Lund O, Nielsen M (2008). NetMHC-3.0: accurate web accessible predictions of human, mouse and monkey MHC class I affinities for peptides of length 8–11. Nucleic Acids Res.

[79] Lundegaard C, Lund O, Nielsen M (2011). Prediction of epitopes using neural network based methods. J Immunol Methods.

[80] Ma Q, Long W, Xing C, Chu J, Luo M, Wang HY, Liu Q, Wang RF (2018). Cancer stem cells and immunosuppressive microenvironment in glioma. Front Immunol.

[81] Majzner RG, Mackall CL (2019). Clinical lessons learned from the first leg of the CAR T cell journey. Nat Med.

[82] Mantovani A, Allavena P, Marchesi F, Garlanda C (2022). Macrophages as tools and targets in cancer therapy. Nat Rev Drug Discov.

[83] Matsushita H, Vesely MD, Koboldt DC, Rickert CG, Uppaluri R, Magrini VJ, Arthur CD, White JM, Chen YS, Shea LK (2012). Cancer exome analysis reveals a T-cell-dependent mechanism of cancer immunoediting. Nature.

[84] Mazzitelli JA, Smyth LCD, Cross KA, Dykstra T, Sun J, Du S, Mamuladze T, Smirnov I, Rustenhoven J, Kipnis J (2022). Cerebrospinal fluid regulates skull bone marrow niches via direct access through dural channels. Nat Neurosci.

[85] Medawar PB (1948). Immunity to homologous grafted skin; the fate of skin homografts transplanted to the brain, to subcutaneous tissue, and to the anterior chamber of the eye. Br J Exp Pathol.

[86] Melnick K, Dastmalchi F, Mitchell D, Rahman M, Sayour EJ (2022). Contemporary RNA therapeutics for glioblastoma. Neuromolecular Med.

[87] Merino DM, McShane LM, Fabrizio D, Funari V, Chen SJ, White JR, Wenz P, Baden J, Barrett JC, Chaudhary R (2020). Establishing guidelines to harmonize tumor mutational burden (TMB): in silico assessment of variation in TMB quantification across diagnostic platforms: phase I of the friends of cancer research TMB harmonization project. J Immunother Cancer.

[88] Nduom EK, Wei J, Yaghi NK, Huang N, Kong LY, Gabrusiewicz K, Ling X, Zhou S, Ivan C, Chen JQ (2016). PD-L1 expression and prognostic impact in glioblastoma. Neuro Oncol.

[89] O'Rourke DM, Nasrallah MP, Desai A, Melenhorst JJ, Mansfield K, Morrissette JJD, Martinez-Lage M, Brem S, Maloney E, Shen A (2017). A single dose of peripherally infused EGFRvIII-directed CAR T cells mediates antigen loss and induces adaptive resistance in patients with recurrent glioblastoma. Sci Transl Med.

[90] Ott PA, Hu Z, Keskin DB, Shukla SA, Sun J, Bozym DJ, Zhang W, Luoma A, Giobbie-Hurder A, Peter L (2017). An immunogenic personal neoantigen vaccine for patients with melanoma. Nature.

[91] Pardoll DM (2012). The blockade of immune checkpoints in cancer immunotherapy. Nat Rev Cancer.

[92] Park JS, Kim IK, Han S, Park I, Kim C, Bae J, Oh SJ, Lee S, Kim JH, Woo DC (2016). Normalization of tumor vessels by Tie2 activation and Ang2 inhibition enhances drug delivery and produces a favorable tumor microenvironment. Cancer Cell.

[93] Piper K, DePledge L, Karsy M, Cobbs C (2021). Glioma stem cells as immunotherapeutic targets: advancements and challenges. Front Oncol.

[94] Pombo Antunes AR, Scheyltjens I, Duerinck J, Neyns B, Movahedi K, Van Ginderachter JA (2020). Understanding the glioblastoma immune microenvironment as basis for the development of new immunotherapeutic strategies. Elife.

[95] Pratt D, Dominah G, Lobel G, Obungu A, Lynes J, Sanchez V, Adamstein N, Wang X, Edwards NA, Wu T (2019). Programmed death ligand 1 is a negative prognostic marker in recurrent isocitrate dehydrogenase-wildtype glioblastoma. Neurosurgery.

[96] Pulous FE, Cruz-Hernández JC, Yang C, Kaya Ζ, Paccalet A, Wojtkiewicz G, Capen D, Brown D, Wu JW, Schloss MJ (2022). Cerebrospinal fluid can exit into the skull bone marrow and instruct cranial hematopoiesis in mice with bacterial meningitis. Nat Neurosci.

[97] Pyonteck SM, Akkari L, Schuhmacher AJ, Bowman RL, Sevenich L, Quail DF, Olson OC, Quick ML, Huse JT, Teijeiro V (2013). CSF-1R inhibition alters macrophage polarization and blocks glioma progression. Nat Med.

[98] Quail DF, Bowman RL, Akkari L, Quick ML, Schuhmacher AJ, Huse JT, Holland EC, Sutton JC, Joyce JA (2016). The tumor microenvironment underlies acquired resistance to CSF-1R inhibition in gliomas. Science.

[99] Quail DF, Joyce JA (2017). The microenvironmental landscape of brain tumors. Cancer Cell.

[100] Raman K (2011). A stochastic differential equation analysis of cerebrospinal fluid dynamics. Fluids Barriers CNS.

[101] Rao G, Latha K, Ott M, Sabbagh A, Marisetty A, Ling X, Zamler D, Doucette TA, Yang Y, Kong LY (2020). Anti-PD-1 induces M1 polarization in the glioma microenvironment and exerts therapeutic efficacy in the absence of CD8 cytotoxic T cells. Clin Cancer Res.

[102] Reardon DA, Brandes AA, Omuro A, Mulholland P, Lim M, Wick A, Baehring J, Ahluwalia MS, Roth P, Bähr O (2020). Effect of nivolumab vs bevacizumab in patients with recurrent glioblastoma: the checkmate 143 phase 3 randomized clinical trial. JAMA Oncol.

[103] Rizvi NA, Hellmann MD, Snyder A, Kvistborg P, Makarov V, Havel JJ, Lee W, Yuan J, Wong P, Ho TS (2015). Cancer immunology. Mutational landscape determines sensitivity to PD-1 blockade in non-small cell lung cancer. Science.

[104] Sampson JH, Gunn MD, Fecci PE, Ashley DM (2020). Brain immunology and immunotherapy in brain tumours. Nat Rev Cancer.

[105] Samstein RM, Lee CH, Shoushtari AN, Hellmann MD, Shen R, Janjigian YY, Barron DA, Zehir A, Jordan EJ, Omuro A (2019). Tumor mutational load predicts survival after immunotherapy across multiple cancer types. Nat Genet.

[106] Schumacher TN, Schreiber RD (2015). Neoantigens in cancer immunotherapy. Science.

[107] Segura-Collar B, Garranzo-Asensio M, Herranz B, Hernández-SanMiguel E, Cejalvo T, Casas BS, Matheu A, Pérez-Núñez Á, Sepúlveda-Sánchez JM, Hernández-Laín A (2021). Tumor-derived pericytes driven by EGFR mutations govern the vascular and immune microenvironment of gliomas. Cancer Res.

[108] Segura-Collar B, Mata-Martínez P, Hernández-Laín A, Sánchez-Gómez P, Gargini R (2022). Blood-brain barrier disruption: a common driver of central nervous system diseases. Neuroscientist.

[109] Shinohara H, Yagita H, Ikawa Y, Oyaizu N (2000). Fas drives cell cycle progression in glioma cells via extracellular signal-regulated kinase activation. Cancer Res.

[110] Simpson AJ, Caballero OL, Jungbluth A, Chen YT, Old LJ (2005). Cancer/testis antigens, gametogenesis and cancer. Nat Rev Cancer.

[111] Slattery K (2019) NK cell metabolism and TGFβ—implications for immunotherapy. In: Gardiner CM (ed), City 10.3389/fimmu.2019.02915 PMC6927492 31921174

[112] Sofroniew MV (2015). Astrocyte barriers to neurotoxic inflammation. Nat Rev Neurosci.

[113] Spranger S, Sivan A, Corrales L, Gajewski TF (2016). Tumor and host factors controlling antitumor immunity and efficacy of cancer immunotherapy. Adv Immunol.

[114] Stone JD, Harris DT, Kranz DM (2015). TCR affinity for p/MHC formed by tumor antigens that are self-proteins: impact on efficacy and toxicity. Curr Opin Immunol.

[115] Sultan H, Salazar AM, Celis E (2020). Poly-ICLC, a multi-functional immune modulator for treating cancer. Semin Immunol.

[116] Takenaka MC, Gabriely G, Rothhammer V, Mascanfroni ID, Wheeler MA, Chao CC, Gutiérrez-Vázquez C, Kenison J, Tjon EC, Barroso A (2019). Control of tumor-associated macrophages and T cells in glioblastoma via AHR and CD39. Nat Neurosci.

[117] Takeshima H, Kuratsu J, Takeya M, Yoshimura T, Ushio Y (1994). Expression and localization of messenger RNA and protein for monocyte chemoattractant protein-1 in human malignant glioma. J Neurosurg.

[118] Tawbi HA, Forsyth PA, Algazi A, Hamid O, Hodi FS, Moschos SJ, Khushalani NI, Lewis K, Lao CD, Postow MA (2018). Combined nivolumab and ipilimumab in melanoma metastatic to the brain. N Engl J Med.

[119] The Cancer Genome Atlas (TCGA) Research Network (2008). Comprehensive genomic characterization defines human glioblastoma genes and core pathways. Nature.

[120] Thorsson V, Gibbs DL, Brown SD, Wolf D, Bortone DS, Ou Yang TH, Porta-Pardo E, Gao GF, Plaisier CL, Eddy JA (2018). The immune landscape of cancer. Immunity.

[121] Tomaszewski W, Sanchez-Perez L, Gajewski TF, Sampson JH (2019). Brain tumor microenvironment and host state: implications for immunotherapy. Clin Cancer Res.

[122] Touat M, Idbaih A, Sanson M, Ligon KL (2017). Glioblastoma targeted therapy: updated approaches from recent biological insights. Ann Oncol.

[123] Touat M, Li YY, Boynton AN, Spurr LF, Iorgulescu JB, Bohrson CL, Cortes-Ciriano I, Birzu C, Geduldig JE, Pelton K (2020). Mechanisms and therapeutic implications of hypermutation in gliomas. Nature.

[124] Tran E, Ahmadzadeh M, Lu YC, Gros A, Turcotte S, Robbins PF, Gartner JJ, Zheng Z, Li YF, Ray S (2015). Immunogenicity of somatic mutations in human gastrointestinal cancers. Science.

[125] Tran E, Turcotte S, Gros A, Robbins PF, Lu YC, Dudley ME, Wunderlich JR, Somerville RP, Hogan K, Hinrichs CS (2014). Cancer immunotherapy based on mutation-specific CD4+ T cells in a patient with epithelial cancer. Science.

[126] Twomey JD, Zhang B (2021). Cancer immunotherapy update: FDA-approved checkpoint inhibitors and companion diagnostics. AAPS J.

[127] Ugel S, De Sanctis F, Mandruzzato S, Bronte V (2015). Tumor-induced myeloid deviation: when myeloid-derived suppressor cells meet tumor-associated macrophages. J Clin Invest.

[128] Venkataramani V, Tanev DI, Strahle C, Studier-Fischer A, Fankhauser L, Kessler T, Körber C, Kardorff M, Ratliff M, Xie R (2019). Glutamatergic synaptic input to glioma cells drives brain tumour progression. Nature.

[129] Venkatesh HS, Johung TB, Caretti V, Noll A, Tang Y, Nagaraja S, Gibson EM, Mount CW, Polepalli J, Mitra SS (2015). Neuronal activity promotes glioma growth through neuroligin-3 secretion. Cell.

[130] Vogelstein B, Papadopoulos N, Velculescu VE, Zhou S, Diaz LA, Kinzler KW (2013). Cancer genome landscapes. Science.

[131] von Roemeling CA, Wang Y, Qie Y, Yuan H, Zhao H, Liu X, Yang Z, Yang M, Deng W, Bruno KA (2020). Therapeutic modulation of phagocytosis in glioblastoma can activate both innate and adaptive antitumour immunity. Nat Commun.

[132] Wang L, Ge J, Lan Y, Shi Y, Luo Y, Tan Y, Liang M, Deng S, Zhang X, Wang W (2020). Tumor mutational burden is associated with poor outcomes in diffuse glioma. BMC Cancer.

[133] Wang Z, Zhang C, Liu X, Sun L, Li G, Liang J, Hu H, Liu Y, Zhang W, Jiang T (2016). Molecular and clinical characterization of PD-L1 expression at transcriptional level via 976 samples of brain glioma. Oncoimmunology.

[134] Watkins S, Robel S, Kimbrough IF, Robert SM, Ellis-Davies G, Sontheimer H (2014). Disruption of astrocyte-vascular coupling and the blood-brain barrier by invading glioma cells. Nat Commun.

[135] Wiendl H, Mitsdoerffer M, Hofmeister V, Wischhusen J, Bornemann A, Meyermann R, Weiss EH, Melms A, Weller M (2002). A functional role of HLA-G expression in human gliomas: an alternative strategy of immune escape. J Immunol.

[136] Wolburg H, Noell S, Mack A, Wolburg-Buchholz K, Fallier-Becker P (2009). Brain endothelial cells and the glio-vascular complex. Cell Tissue Res.

[137] Xie G, Dong H, Liang Y, Ham JD, Rizwan R, Chen J (2020). CAR-NK cells: a promising cellular immunotherapy for cancer. EBioMedicine.

[138] Xue S, Song G, Yu J (2017). The prognostic significance of PD-L1 expression in patients with glioma: a meta-analysis. Sci Rep.

[139] Yan Y, Zeng S, Gong Z, Xu Z (2020). Clinical implication of cellular vaccine in glioma: current advances and future prospects. J Exp Clin Cancer Res.

[140] Yin J, Kim SS, Choi E, Oh YT, Lin W, Kim TH, Sa JK, Hong JH, Park SH, Kwon HJ (2020). ARS2/MAGL signaling in glioblastoma stem cells promotes self-renewal and M2-like polarization of tumor-associated macrophages. Nat Commun.

[141] Yin W, Jiang X, Tan J, Xin Z, Zhou Q, Zhan C, Fu X, Wu Z, Guo Y, Jiang Z (2020). Development and validation of a tumor mutation burden-related immune prognostic model for lower-grade glioma. Front Oncol.

[142] Zeng J, Zhang XK, Chen HD, Zhong ZH, Wu QL, Lin SX (2016). Expression of programmed cell death-ligand 1 and its correlation with clinical outcomes in gliomas. Oncotarget.

[143] Zhang D, Qiu B, Wang Y, Guan Y, Zhang L, Wu A (2017). Temozolomide increases MHC-I expression via NF-κB signaling in glioma stem cells. Cell Biol Int.

[144] Zhao J, Chen AX, Gartrell RD, Silverman AM, Aparicio L, Chu T, Bordbar D, Shan D, Samanamud J, Mahajan A (2019). Immune and genomic correlates of response to anti-PD-1 immunotherapy in glioblastoma. NatMed.

[145] Zhao Q, Laverdure JP, Lanoix J, Durette C, Côté C, Bonneil É, Laumont CM, Gendron P, Vincent K, Courcelles M (2020). Proteogenomics uncovers a vast repertoire of shared tumor-specific antigens in ovarian cancer. Cancer Immunol Res.

[146] Zhao W, Zhao G, Wang B (2018). Revisiting GM-CSF as an adjuvant for therapeutic vaccines. Cell Mol Immunol.

