# Correction to: Advanced immunotherapies for glioblastoma: tumor neoantigen vaccines in combination with immunomodulators

**DOI:** 10.1186/s40478-023-01600-2

**Published:** 2023-07-12

**Authors:** Berta Segura-Collar, Sara Hiller-Vallina, Olaya de Dios, Marta Caamaño-Moreno, Lucia Mondejar-Ruescas, Juan M. Sepulveda-Sanchez, Ricardo Gargini

**Affiliations:** 1 Instituto de Investigaciones Biomédicas I+12, Hospital Universitario, 12 de Octubre, Madrid, 28041 Spain; 2 Pathology and Neurooncology Unit, Hospital Universitario, 12 de Octubre, Av. de Córdoba, S/N, Madrid, 28041 Spain; 3 Instituto de Salud Carlos III, UFIEC, 28222 Majadahonda, Spain; 4 Medical Oncology, Hospital Universitario, 12 de Octubre, 28041 Madrid, Spain

Acta Neuropathologica Communications (2023) 11:79

10.1186/s40478-023-01569-y

Following publication of the original article [1], the authors reported incorrect information in the ‘Acknowledgements’ section.

The first part of the ‘Acknowledgements’ section originally read: Work was supported by Ministerio de Ciencia e Innovación and FEDER funds: CP21/00116 and PI22/01171 to RG.

The sentence should read: This study has been funded by Instituto de Salud Carlos III (ISCIII) through the project “CP21/00116 and PI22/0117” and co-funded by the European Union to RG.

The original article [1] has been updated.
